# Supplementary material for: Support and follow-up needs of patients discharged from intensive care after severe COVID-19: a mixed-methods study of the views of UK general practitioners and intensive care staff during the pandemic’s first wave
Source: BMJ Open. 2021 May 11;11(5):e048392. doi: 10.1136/bmjopen-2020-048392 (PMC8117472; doi:10.1136/bmjopen-2020-048392)
Supplement: Supplementary data [file bmjopen-2020-048392supp001.pdf]

Supplementary material

Version 1.0 (12/05/2020)

THE UNIVERSITY of York

Post-ICU support for patients discharged after COVID 19: Participant Information Sheet

We would like to invite you to take part in a research study. Before you decide, it is important for you to understand why this research is being done and what it would involve for you. Please take time to read the following information carefully and discuss it with others if you wish. Ask us if there is anything that is not clear or if you would like more information. Take time to decide whether or not you wish to take part.

Thank you for reading this.

What is the purpose of the study?

Evidence shows that patients discharged following an Intensive Care Unit (ICU) stay may experience significant physical, mental and cognitive problems after leaving the unit and once they return to their homes and communities. These may persist even five years after leaving hospital[1] and a recent study shows that six months post-discharge, 25% of survivors suffer severe disability, 22% have depression or anxiety and 40% have returned to work.[2]

The COVID-19 pandemic represents clear challenges, with follow-up and transition post-ICU stay likely to have been unavoidably curtailed. This research project is part of a wider study looking at the availability and form of follow-up services available for ICU survivors of COVID-19. This interview study will ask a sample of ICU leads and critical care staff about their views around the future needs of patients post-ICU discharge following severe COVID illness and early reflections on ICU care and transitions out of ICU during the COVID-19 outbreak.

Why am I being asked to take part?

As part of the project we are interviewing ICU leads and critical care staff to explore views and experiences around the needs of COVID patients, post-ICU discharge. You have been selected to represent one of these perspectives and we are keen to talk to you more about this.

Do I have to take part?

No, it is entirely up to you to decide whether you would like to take part. If you have any questions about taking part you can talk to a member of the research team. Even if you have agreed to take part, you are free to withdraw from the study at any time, without giving a reason.

What will happen to me if I take part?

If you decide that you would like to take part in the study you will be sent a consent form electronically to read, which will be discussed prior to interview. We will then ask you to give verbal consent during the telephone or videoconference interview.

A researcher will approach you to arrange a time to conduct the interview that best suits you. Due to the current need for social distancing, interviews will take place remotely either by telephone or videoconference call. The interview will last up around 30 minutes and will be audio-recorded (with your permission), and sections transcribed for analysis.

---

[1] Herridge et al (2011) Functional Disability 5 Years after Acute Respiratory Distress Syndrome. N Engl J Med; 364:1293-1304. DOI: 10.1056/NEJMoa1011802
[2] Hodgson et al (2017) The impact of disability in survivors of critical illness. Intensive Care Med.;43(7):992-1001. DOI: 10.1007/s00134-017-4830-0

Version 1.0 (12/05/2020)

You will be given a unique study number for the duration of the study so that your name and organisation will not be used in any publications and will not be made available outside the research team.

What are the possible benefits of taking part?

Whilst there are no personal benefits to you for taking part, the findings of this study will develop a better understanding of the future needs of COVID 19 patients discharged from ICU.

Expenses and payments

This study is funded by the NIHR Policy Research Programme and we will arrange interviews at a time convenient to you so that no costs will be associated with you taking part in the study. Thus, no participant expenses have been allocated for this study.

What will happen to data that are collected about me?

We will remove all names and other identifying information before the data are analysed and results presented. Any records that identify you will be held separately to the other information we collect and your data will be held in a secure location, in accordance with the General Data Protection Regulations (GDPR) and the Data Protection Act 2018. Only researchers that are part of the research team in York and The King's Fund will have access to the data. Data will be stored for 5 years, to enable analysis and publication and will then be destroyed. If you decide to change your mind about taking part in the study, you can request that the data collected be destroyed. Following this, your data will not be analysed or used in the report of the findings.

Who has reviewed this study?

This study has been reviewed by the University of York's Department of Health Sciences' Research Governance Committee.

Who is organising and funding this research?

The research is funded by the NIHR Policy Research Programme. The research funding covers only the costs of undertaking the research; researchers will not receive payment for conducting the study. Findings will be reported in aggregated form, interviewees and organisations will not be named or otherwise identifiable when findings are reported.

Who can I contact for more information?

If you have any queries about the study please contact:		If you need to make a complaint or speak to someone independent, please contact:
Dr Laura Jefferson<br>Research Fellow<br>Department of Health Sciences<br>Area 4 Seebohm Rowntree Building<br>University of York<br>Heslington<br>York YO10 5DD<br><br>Email: laura.jefferson@york.ac.uk		Prof Patrick Doherty<br>Chair of Research Committee<br>Department of Health Sciences<br>Area 4 Seebohm Rowntree Building<br>University of York<br>Heslington<br>York<br><br>Email: patrick.doherty@york.ac.uk

Thank you for reading this information sheet and for considering whether to take part in this study.

Version 1.0 (12/05/2020)

THE UNIVERSITY of York

CONSENT FORM

Participant Identification Number: ☐☐☐☐

Title of study: Post-ICU support for patients discharged after COVID 19: interviews with critical care staff

Please initial the boxes to confirm verbal consent given

1. I confirm that I have read and understand the information sheet version [1] dated [12/05/20] for the above study. I have had the opportunity to consider the information, ask questions and have had these answered satisfactorily. ☐

2. I understand that my participation is voluntary and that I am free to withdraw at any time without giving reason. ☐

3. I agree to this consent form and other data collected as part of this research study being kept at the University of York and The King's Fund. ☐

4. I understand that relevant sections of data collected during the study may be looked at by individuals from the University of York and The King's Fund and from regulatory authorities. I give permission for these individuals to have access to these records. ☐

6. I agree to the interviews being audio recorded and sections transcribed. ☐

7. I understand that direct quotations may be used in publications but no information will be released or printed that would identify me. ☐

8. I understand and agree that the research team will securely store my identifiable details in order to contact me in future regarding this study, or other related studies (e.g. telephone/text/email). Identifiable details, including a copy of the consent form, will be available only to the research team, other than for purposes of monitoring and audit. ☐

9. I agree to take part in the above study. ☐

______________________ ______________________

Name of participant *(please print)* Date verbal consent given

______________________ ______________________

Name of person taking consent *(please print)* Date

Version 1.0 (29/04/2020)

THE UNIVERSITY *of York*

Post-ICU support for patients discharged after COVID 19: Participant Information Sheet

We would like to invite you to take part in a research study. Before you decide, it is important for you to understand why this research is being done and what it would involve for you. Please take time to read the following information carefully and discuss it with others if you wish. Ask us if there is anything that is not clear or if you would like more information. Take time to decide whether or not you wish to take part.

Thank you for reading this.

What is the purpose of the study?

Evidence shows that patients discharged following an Intensive Care Unit (ICU) stay may experience significant physical, mental and social problems after leaving the unit and once they return to their homes and communities. These may persist even five years after leaving hospital[1] and a recent study shows that six months post-discharge, 25% of survivors suffer severe disability, 22% have depression or anxiety and 40% have returned to work.[2]

The COVID-19 pandemic represents clear challenges, with follow-up and transition post-ICU stay likely to have been unavoidably curtailed. This research project is part of a wider study looking at the availability and form of follow-up services available for ICU survivors of COVID-19. This interview study will explore GPs experience of managing patients following an ICU stay in general and those patients discharged following severe COVID illness. We will also identify any information needs amongst GPs in relation to patients recovering from severe COVID illness.

Why am I being asked to take part?

As part of the project we are interviewing GPs to explore how patients are managed in general practice following discharge from ICU, in general and following severe COVID illness. You have been selected to represent one of these perspectives and we are keen to talk to you more about this.

Do I have to take part?

No, it is entirely up to you to decide whether you would like to take part. If you have any questions about taking part, you can talk to a member of the research team. Even if you have agreed to take part, you are free to withdraw from the study at any time, without giving a reason.

What will happen to me if I take part?

If you decide that you would like to take part in the study you will be sent a consent form electronically to read, which will be discussed prior to interview. We will then ask you to give verbal consent during the telephone or videoconference interview.

A researcher will approach you to arrange a time to conduct the interview that best suits you. Due to current social distancing restrictions, interviews will take place remotely either by telephone or videoconference call. The interview will last up around 30 minutes and will be used to find out more information about your experiences of managing patients following usual

[1] Herridge et al (2011) Functional Disability 5 Years after Acute Respiratory Distress Syndrome. N Engl J Med; 364:1293-1304. DOI: 10.1056/NEJMoa1011802

[2] Hodgson et al (2017) The impact of disability in survivors of critical illness. Intensive Care Med.;43(7):992-1001. DOI: 10.1007/s00134-017-4830-0

discharge from ICU, and discharge following severe COVID illness. The interview will be audio-recorded, with your permission, and sections may be transcribed for analysis.

You will be given a unique study number for the duration of the study so that your name and organisation will not be used in any publications and will not be made available outside the research team.

What are the possible benefits of taking part?

Whilst there are no personal benefits to you for taking part, the findings of this study will develop a better understanding of the practices being used to care for COVID 19 patients discharged from ICU and any informational needs within general practice.

Expenses and payments

This study is funded by the NIHR Policy Research Programme and we will arrange interviews at a time convenient to you so that no costs will be associated with you taking part in the study. Thus, no participant expenses have been allocated for this study.

What will happen to data that are collected about me?

We will remove all names and other identifying information before the data are analysed and results presented. Any records that identify you will be held separately to the other information we collect, and your data will be held in a secure location, in accordance with the General Data Protection Regulations (GDPR) and the Data Protection Act 2018. Only researchers that are part of the research team in York and The King's Fund will have access to the data. Data will be stored for 5 years, to enable analysis and publication and will then be destroyed. If you decide to change your mind about taking part in the study, you can request that the data collected be destroyed. Following this, your data will not be analysed or used in the report of the findings.

Who has reviewed this study?

This study has been reviewed by the University of York's Department of Health Sciences' Research Governance Committee.

Who is organising and funding this research?

The research is funded by the NIHR Policy Research Programme. The research funding covers only the costs of undertaking the research; researchers will not receive payment for conducting the study. Findings will be reported in aggregated form, interviewees and organisations will not be named or otherwise identifiable when findings are reported.

Who can I contact for more information?

If you have any queries about the study please contact:<br><br>Dr Laura Jefferson<br>Research Fellow<br>Department of Health Sciences<br>Area 4 Seebohm Rowntree Building<br>University of York<br>Heslington<br>York YO10 5DD<br><br>Email: laura.jefferson@york.ac.uk		If you need to make a complaint or speak to someone independent, please contact:<br><br>Prof Patrick Doherty<br>Chair of Research Committee<br>Department of Health Sciences<br>Area 4 Seebohm Rowntree Building<br>University of York<br>Heslington<br>York<br><br>Email: patrick.doherty@york.ac.uk

Thank you for reading this information sheet and for considering whether to take part in this study.

Version 1.0 (29/04/2020)

THE UNIVERSITY of York

CONSENT FORM

Participant Identification Number: ☐☐☐☐

Title of study: Post-ICU support for patients discharged after COVID 19: interviews with GPs

Please initial the boxes to confirm verbal consent given

1. I confirm that I have read and understand the information sheet version [1] dated [29/04/20] for the above study. I have had the opportunity to consider the information, ask questions and have had these answered satisfactorily. ☐

2. I understand that my participation is voluntary and that I am free to withdraw at any time without giving reason. ☐

3. I agree to this consent form and other data collected as part of this research study being kept at the University of York and The King's Fund. ☐

4. I understand that relevant sections of data collected during the study may be looked at by individuals from the University of York and The King's Fund and from regulatory authorities. I give permission for these individuals to have access to these records. ☐

6. I agree to the interviews being audio recorded and sections transcribed. ☐

7. I understand that direct quotations may be used in publications but no information will be released or printed that would identify me. ☐

8. I understand and agree that the research team will securely store my identifiable details in order to contact me in future regarding this study, or other related studies (e.g. telephone/text/email). Identifiable details, including a copy of the consent form, will be available only to the research team, other than for purposes of monitoring and audit. ☐

9. I agree to take part in the above study. ☐

____________________	____________________
Name of participant *(please print)*	Date verbal consent given
____________________	____________________
Name of person taking consent *(please print)*	Date
