# Supplementary material for: Support and follow-up needs of patients discharged from intensive care after severe COVID-19: a mixed-methods study of the views of UK general practitioners and intensive care staff during the pandemic’s first wave
Source: BMJ Open. 2021 May 11;11(5):e048392. doi: 10.1136/bmjopen-2020-048392 (PMC8117472; doi:10.1136/bmjopen-2020-048392)
Supplement: Author's manuscript [file bmjopen-2020-048392.draft_revisions.pdf]

BMJ Open is committed to open peer review. As part of this commitment we make the peer review history of every article we publish publicly available.

When an article is published we post the peer reviewers' comments and the authors' responses online. We also post the versions of the paper that were used during peer review. These are the versions that the peer review comments apply to.

The versions of the paper that follow are the versions that were submitted during the peer review process. They are not the versions of record or the final published versions. They should not be cited or distributed as the published version of this manuscript.

BMJ Open is an open access journal and the full, final, typeset and author-corrected version of record of the manuscript is available on our site with no access controls, subscription charges or pay-per-view fees (http://bmjopen.bmj.com).

If you have any questions on BMJ Open's open peer review process please email info.bmjopen@bmj.com

BMJ Open

Support and follow-up needs of patients discharged from Intensive Care after severe COVID-19: a mixed-methods study of the views of UK general practitioners and intensive care staff during the pandemic's first wave

Journal:	*BMJ Open*
Manuscript ID	bmjopen-2020-048392
Article Type:	Original research
Date Submitted by the Author:	04-Jan-2021

Complete List of Authors:	Castro-Avila, Ana; University of York, Department of Health Sciences; Universidad del Desarrollo Facultad de Medicina Clínica Alemana, Carrera de Kinesiología<br>Jefferson, Laura; University of York, Department of Health Sciences<br>Dale, Veronica; University of York, Health Sciences<br>Bloor, Karen; University of York, Department of Health Sciences

Keywords:	COVID-19, INTENSIVE & CRITICAL CARE, REHABILITATION MEDICINE

SCHOLARONE™
Manuscripts

BMJ

I, the Submitting Author has the right to grant and does grant on behalf of all authors of the Work (as defined in the below author licence), an exclusive licence and/or a non-exclusive licence for contributions from authors who are: i) UK Crown employees; ii) where BMJ has agreed a CC-BY licence shall apply, and/or iii) in accordance with the terms applicable for US Federal Government officers or employees acting as part of their official duties; on a worldwide, perpetual, irrevocable, royalty-free basis to BMJ Publishing Group Ltd ("BMJ") its licensees and where the relevant Journal is co-owned by BMJ to the co-owners of the Journal, to publish the Work in this journal and any other BMJ products and to exploit all rights, as set out in our licence.

The Submitting Author accepts and understands that any supply made under these terms is made by BMJ to the Submitting Author unless you are acting as an employee on behalf of your employer or a postgraduate student of an affiliated institution which is paying any applicable article publishing charge ("APC") for Open Access articles. Where the Submitting Author wishes to make the Work available on an Open Access basis (and intends to pay the relevant APC), the terms of reuse of such Open Access shall be governed by a Creative Commons licence – details of these licences and which Creative Commons licence will apply to this Work are set out in our licence referred to above.

Other than as permitted in any relevant BMJ Author's Self Archiving Policies, I confirm this Work has not been accepted for publication elsewhere, is not being considered for publication elsewhere and does not duplicate material already published. I confirm all authors consent to publication of this Work and authorise the granting of this licence.

For peer review only

Support and follow-up needs of patients discharged from Intensive Care after severe COVID-19: a mixed-methods study of the views of UK general practitioners and intensive care staff during the pandemic's first wave

Ana Castro-Avila, Laura Jefferson, Veronica Dale, Karen Bloor

Department of Health Sciences, University of York, Heslington, YO10 5DD

Correspondence to A Castro-Avila ana.castro@york.ac.uk Area 4 Seebohm Rowntree Building, Department of Health Sciences, University of York, Heslington, YO10 5DD.

Keywords: Critical Illness/rehabilitation, Intensive care units, survivors, COVID-19

Word count: 3749

For peer review only

Abstract

Objectives: To identify follow-up services planned for COVID-19 patients discharged from ICU, and to explore the views of ICU staff and General Practitioners (GPs) regarding these patients' future needs and care coordination.

Setting: GPs surgeries and Acute NHS Trusts in the UK

Participants: GPs and ICU leads treating patients discharged from ICU.

Primary and secondary outcomes: Survey responses and interviews exploring usual follow-up practice after ICU discharge and changes in follow-up during the pandemic, and GP awareness of follow-up and support needs of patients discharged from ICU.

Results: We obtained 170 survey responses and conducted 23 interviews. Over 60% of GPs were unaware of the follow-up services generally provided by their local hospitals, and whether or not these were functioning during the pandemic. Eighty percent of ICUs reported some form of follow-up services, with 25% of these suspending provision during the peak of the pandemic, and over half modifying their provision (usually to provide the service remotely). Complex funding streams, poor communication between primary and secondary care, and lack of clarity about who was responsible for referrals and follow-up were the most common problems identified. Discharge documentation was described as poor and lacking key information. Both groups mentioned difficulties accessing services in the community.

Conclusions: The pandemic has highlighted long-standing issues of continuity of care and complex funding streams for post-ICU follow-up care. The large cohort of ICU patients admitted due to COVID-19 highlights the need for improved follow-up services and communication between specialists and GPs, not only for COVID-19 patients, but for all those discharged from ICU.

Article Summary

Strengths and limitations of this study

- This is the first study exploring NHS staff views of post-ICU follow-up services to support patients recovering from severe COVID-19.
- Responses to our survey had good geographic spread but were limited in number and relied on volunteers.
- GP interviews were small in number, but our findings align with those of larger studies conducted before the pandemic.

For peer review only

Introduction

The significant physical, mental and cognitive problems patients face following a stay in an Intensive Care Unit (ICU) are well-documented.[1 2] Including prolonged muscle weakness, cognitive dysfunction and symptoms of post-traumatic stress disorder (PTSD), these are often collectively referred to as 'post intensive care syndrome' (PICS)[3] and may persist even five years after leaving hospital.[4-7] Six months post-discharge, 25% of survivors suffer severe disability,[8] and only around 55% have returned to work.[9] Psychological disorders including depression, anxiety, and post-traumatic stress disorder (PTSD) are common, affecting 55% in the first year following ICU discharge.[10] The variety and severity of sequelae vary substantially.

The COVID-19 pandemic has resulted in a large and rapid increase in intensive care activity, which will challenge post-ICU services in several ways. Increases in ICU capacity necessitated the use of less experienced staff and very high workloads. More stringent infection control protocols created new potential causes of distress, including not allowing family members inside the unit and health care professionals wearing Personal Protective Equipment (PPE). These factors might lead to a very large cohort of critical illness survivors that might have greater than expected needs due to the context and length of their critical care stay, [11] which could put the current capacity of services under stress.

Post-ICU follow-up from hospital teams is likely to have been compromised during the pandemic. The availability, form and scale of services for ICU survivors of COVID-19 are largely unknown, and patient needs are difficult to predict. Timely, appropriate support could potentially prevent future problems in patients' physical, cognitive and mental health and care. This study aims to identify follow-up services that were available during and after the UK's first wave, early reflections on care during the first wave and the views of critical care staff and GPs regarding the future needs of COVID-19 patients.

This study aims to identify follow-up services planned for COVID-19 patients discharged from ICU, and to explore the views of ICU staff and General Practitioners (GPs) regarding these patients' future needs and care coordination.

Methods

We employed mixed methods including online surveys and semi-structured telephone interviews with ICU follow-up lead clinicians and general practitioners (GPs).

In collaboration with clinicians in the field, we developed a very brief questionnaire of ICU staff (Table 1) to determine usual procedures of follow-up of patients after ICU discharge, and changes during the pandemic. The Intensive Care Society, Faculty of Intensive Care Medicine, British Association of Critical Care Nurses, and leading experts on intensive care disseminated this survey through newsletters, targeted emails and Twitter. We invited clinicians managing follow-up care of discharged ICU patients to participate, and asked respondents to volunteer for interviews. We sampled volunteers purposively by geographical location, need for expanded bed capacity during the pandemic, and changes implemented.

In collaboration with the Royal College of General Practitioners (RCGP), we developed and distributed a very brief questionnaire (Table 2) exploring GPs' awareness of post-ICU follow-up services, and broad concerns about severe COVID-19 patients' care. RCGP also included three of these questions in a routine survey of their GP research panel. GPs proved difficult to recruit to interviews through the survey, so we supplemented this with 'snowballing' using contacts at the

For peer review only - http://bmjopen.bmj.com/site/about/guidelines.xhtml

RCGP, University of York and The King's Fund. We attempted to generate geographical spread in terms of location and COVID-19 incidence.

Both surveys were piloted with clinicians and experts to ensure clarity, conciseness, precision of language, and identify any essential omissions. We used Qualtrics XM software (Qualtrics, Provo, UT) to distribute the surveys and collect responses.

We conducted 30-minute semi-structured telephone interviews with GPs and ICU leads, which were audio-recorded for analysis. We asked for consent verbally before the start of each interview. Interviews with ICU leads explored views on whether and how the future needs of COVID-19 patients differed from non-COVID patients and captured early reflections on ICU care and transitions during the first wave of the COVID-19 outbreak. Interviews with GPs explored their prior experience of managing post-ICU patients, and information needs in relation to severe COVID-19.

Data analysis

Quantitative

Survey data were exported to IBM SPSS Statistics for Windows (Version 26. Armonk, NY: IBM Corp) for analysis. Absolute and relative frequencies were used to summarise responses. We calculated the average rank for the question about GPs' concerns regarding future care needs of patients recovering from a COVID-19 related critical care stay.

Qualitative

We conducted thematic analysis to synthesise the results and produce insights using NVivo (version 12. QSR International Pty Ltd.) As is increasingly being adopted in rapid qualitative research,[12] analysis was undertaken directly from audio-recordings and detailed notes, transcribing sections for use as quotations. Two researchers (ACA and LJ) coded, each producing an initial framework with main themes, which were discussed with the wider team and topic experts to refine the framework and distil overarching themes. Some representative quotes are presented to contextualise and aid interpretation.

Patient and Public Involvement (PPI)

We reviewed and discussed the project protocol with our PPI group, refining it in response. We were, however, unable to capture patients' views on follow-up services in our study timescale.

Results

Surveys

Between 15th June and 3rd August, ICU follow-up lead clinicians from 112 units (43% of Acute NHS Trusts in England) responded to our survey. 83% were based in England, and 96% were from mixed intensive care and high dependency units. On average, units more than doubled bed capacity at the height of the first wave (Table 1).

Follow-up services were offered in 80 units (71% of those sampled); of those, 20 reported ceasing provision and 53 modifying provision of services during the pandemic. Eight units implemented a new follow-up service after the peak of the pandemic. Provision of occupational therapy and physiotherapy were the services with the greatest increase (Table 1).

For peer review only

Table 1 Responses of ICU leads about follow-up services during the pandemic

Information about the unit	n=112	Responses		
When is the critical care discharge summary sent to the patient's General Practitioner?	38	When the patient is discharged from hospital		
	36	After critical care discharge, but before discharge from hospital		
	16	Other		
	8	I do not know		
When is the first follow-up?	29	2-3 months after discharge from hospital		
	28	2-3 months after discharge from critical care		
	11	Other		
	8	4-6 months after discharge from hospital		
	3	1 month after discharge from hospital		
Number of beds in your unit, mean (SD)	93	Before: 13.9 (11.1)		
		During peak: 33.7 (31.0)		
		Change: 20.1 (23.9)		
**Changes reported**	**n=53**	**Details of change**		
Change in the format of the contacts (e.g. remote consultations)	39	Remote consultations via telephone or video call		
	15	Face-to-face clinics in hospital wearing personal protective equipment		
	2	Home visits		
Change in the number of professionals involved, mean (SD)	22	Before: 2.8 (1.9)		
		After: 4.1 (2.4)		
		Change: 1.3 (2.6)		
Change in the timing of the first contact	24	Time before first follow-up contact is shorter than usual		
	2	Time before first follow-up contact is longer than usual		
Services available	25		Before	During
		Review of ICU history/diary and ICU events with patient	23	22
		Assessment of sleep	15	12
		Physiotherapy	13	17
		Medicines reconciliation	10	8
		Psychology	9	10
		Assessment of sexual function	8	4
		Dietetics	6	5
		Speech and Language Therapy	5	6
		Cognitive assessment,	5	4
		Psychiatry,	2	2
		Social work	2	0
		Occupational Therapy,	1	5

58 GPs responded to our survey and an additional 537 responded to three questions distributed by RCGP (Table 2). 78% of RCGP responses came from England, 61% were female, 63% were 35-54 years old, and 83% were white.

Interview findings

We conducted 23 interviews between 23rd June and 30th July; 17 with ICU staff (7 ICU consultants, 7 senior nurses, 3 rehabilitation coordinators) and 6 GPs. ICU interviews covered all UK regions, with the ICUs having an average capacity before the pandemic of 14 beds (range 4-60); increasing by 16 beds on average (range 2-38 beds). The GPs covered different regions of England and a mix of patient demographics.

For peer review only

Table 2 Responses from GPs survey

Question	Responses	Our sample	RCGP
Does your nearest Hospital Trust have specific follow-up services for all patients who have been discharged from critical care?	n	58	-
	I do not know	36 (62%)	-
	Yes	11 (19%)	-
	No	11 (19%)	-
Is the follow-up service functioning during the COVID-19 pandemic?	n	45	-
	I do not know	39 (87%)	-
	Yes	6 (13%)	-
Within your patient list, are you aware of any patients who have required critical care for severe COVID-19?	n	56	537
	Yes	33 (59%)	208 (39%)
	No	13 (23%)	244 (45%)
	I do not know	10 (18%)	85 (16%)
How many of your patients went through critical care due to severe COVID-19?	n	24	462
	Mean (min-max)	4.4 (1-20)	4.4 (0-50)
Considering future patients in your practice recovering from a COVID-19 related critical care stay, please rank your concerns about their care, mean rank (SD)	n	40	447
	Physical health care	1.9 (1.4)	1.4 (1.3)
	Mental Health care	2.4 (1.1)	1.4 (1.2)
	Access to rehabilitation services	3.1 (1.3)	1.6 (1.3)
	Cognitive functioning	3.5 (1.2)	1.8 (1.3)
	Access to social care	4.0 (1.1)	1.7 (1.4)

The ICU environment

All interviewees reported opening new areas and bringing nurses from other areas (e.g. theatre, surgical recovery, other hospital wards) particularly those with ICU training. Consultants increased the frequency of their rotations to ensure continuous coverage. Administrative tasks for clinicians were suspended and all staff providing outpatient or outreach services returned to inpatient activities. ICU nurses split their time between patient care, staff supervision and training new staff, which was reported to increase workload and stress. Hospitals with greater bed capacity implemented proning and intubating teams, and some implemented retrieval teams to transfer patients between hospitals.

ICU staff reported that PPE was uncomfortable and created difficulties in building relationships with patients, hindering instructions and explanations. Visiting of patients' family members was suspended, except at end of life; therefore, staff implemented different strategies to update families and enable virtual visiting.

Numerous ICU interviewees mentioned that patients with COVID-19 may represent a new patient group, but are still ICU survivors, with the weakness, mental and cognitive problems these patients commonly suffer. They expected COVID-19 patients to suffer a longer-lasting deterioration of lung function, potential issues with renal function, a high incidence of shoulder injuries due to proning, and cognitive problems related to the incidence of delirium.

Some thought it was too early to tell whether they will experience more physiological and psychological problems, but many highlighted particular treatments, including prolonged and deep sedation, opioids and neuromuscular blockers, which are associated with increased risk of muscular weakness, polyneuropathy, and cognitive impairments. Patients experienced extended periods in a prone position, mechanical ventilation and less experienced nursing staff. One consultant believed that actively screening for mental health problems was needed (ICUcons09, Scotland).

For peer review only

For peer review only - http://bmjopen.bmj.com/site/about/guidelines.xhtml

One ICU nurse had administered a mental health questionnaire with COVID-19 patients as part of patient audit, reporting that ventilated patients had the same psychological issues as other ICU patients, but those who received continuous positive airways pressure (CPAP) and were therefore conscious, had worse scores. An ICU consultant echoed this and also highlighted potential difficulties due to PPE:

> *"The other people in the bay watched [another patient] die over a number of days… It doesn't surprise me that the people here perhaps, more awake and aware are very, very traumatised by the experiences […] [Some patients] have delusional thoughts. I mean, I think that's gonna be a lot worse when you're surrounded by someone wearing a hazmat suit." ICUcons06, South West.*

Provision of follow-up services

Before COVID-19, most ICU interviewees reported having a post-ICU follow-up service; the few that did not were planning to implement one after the pandemic. Most follow-up services were suspended during the peak of the first wave, as staff returned to in-hospital clinical duties. The few places that continued to provide such services used telephone follow-up, delivered by staff that were shielding.

Reported provision varies greatly, with some units delivering follow-up with just a consultant and/or a senior nurse, while others have multidisciplinary teams. Some units start their follow-up during the ICU stay, and have designated professionals to assess, refer and follow patients during the hospital episode and into the community. Others with well-established follow-up services refer ICU patients to pulmonary or cardiac rehab to recover fitness and muscle strength.

All unit staff we interviewed follow patients up 2-3 months after ICU discharge, but a minority also routinely call patients weekly (ICUnurse04, North East) or monthly (ICUnurse08, East Midlands). All had to change the format of their follow-up during the pandemic, most replaced clinics with telephone calls or virtual consultations. One senior nurse highlighted the challenges of reduced non-verbal communication and time-limited calls:

> *"Phone calls don't really cut it because unless you're very skilled at talking to people, assessing people, you're not going to pick up on all those cues that people give out […] if we've got half an hour appointment, we won't get much out from in 10 minutes, but they'll open up" ICUnurse14, East of England*

Two ICU interviewees said that they were implementing separate clinics for COVID-19 patients to carry out extra recommended assessments, such as a chest x-ray at 6 weeks post-discharge as recommended by the British Thoracic Society.[13]

In some locations COVID-19 rehab hospitals have been set up to provide specialist care and a "step down" for patients *"that are not quite well enough to leave the acute setting and not quite well enough to go home"* (GP1003, Yorkshire). This provided the opportunity for expert care to be delivered but relied on CCG funding and *"proactive planning for the worst-case scenario"* (GP1003, Yorkshire).

GPs were concerned about the complex psychological needs of patients recovering from severe COVID and that greater emphasis is placed on the physical needs of patients, with insufficient consideration of psychological support. All ICU interviewees agreed about the need for increased psychological support services.

For peer review only - http://bmjopen.bmj.com/site/about/guidelines.xhtml

Some ICU interviewees questioned the capacity of community rehabilitation services to improve patients' functioning. Both GPs and ICU staff felt that previous notions of thresholds for functional status post-ICU (several commented on assessments of patients climbing a flight of stairs) were arbitrary and not suitable for the wider age group of patients affected by COVID-19.

> *"Community Services work at getting someone functional. They don't work at getting them back to the state that they were at before they came into hospital. So, considering that a lot of our patients were younger patients, walking with a Zimmer frame to and from a bathroom aren't really what they want to be doing. They want to be getting back to their fitness level and back to work." ICUrehab15, Wales*

One GP commented that: *"it's only once they are home that the true level of need is understood"* (GP1005, North West); at this point primary care and community services need to step in, but the support needs of these patients may be beyond their expertise and the capacity of services.

Some GP practices had taken proactive steps to follow-up patients discharged from ICU (before and since COVID-19). One practice, pre-COVID-19, developed a list of 'at risk' patients that was monitored by a nurse practitioner and discussed at daily practice meetings, with the aim of reducing hospital admissions. One London practice had instigated weekly follow-up calls with COVID-19 patients discharged from ICU, following a 'near miss' event whereby a serious complication had been detected opportunistically during a GP follow-up call.

Barriers to provision of follow-up services

Both ICU staff and GPs found referrals to community follow-up services difficult, with differences in opinion about whose responsibility this was, as well as problems with waiting lists (particularly for mental health services). Community rehabilitation services were described as *"patchy"* (ICUnurse08, East Midlands). Key barriers expressed relate to funding complexities, remit and expertise, and communication.

ICU interviewees in England felt the lack of a tariff for funding ICU follow-up clinics created variation in service provision. Both ICU and GP interviewees believed that community teams similar to those for stroke or cardiac rehabilitation should be set up for post-ICU patients.

Several interviewees were concerned that already overstretched community services with existing waiting lists could not meet the increase in demand from COVID-19 without improved funding and infrastructure. One GP described the closure of some community services, and ICU interviewees had concerns that those discharged from ICU in the peak of the pandemic did not have anywhere to go.

> *"We've had patients in tears, we've had seven patients through telephone calls. And all of them are absolutely distraught and feel like they've been abandoned in the community […] because they were thrown out of hospital very quickly. There's no services in the community for them at all." ICUrehab15, Wales*

Both ICU and GP interviewees felt that hospital services were better placed to follow-up patients discharged from ICU, because they have a better understanding of the patient's needs. There appears, however, to be a general lack of awareness about the difficulties of coordinating patients' needs in each setting.

> *"I'm not sure the hospitals are always very aware of what services are available in the community… To give you a COVID example, I had a doctor ring me up and say*

For peer review only - http://bmjopen.bmj.com/site/about/guidelines.xhtml

> *'he's been in hospital for a long time, can you make sure he sees a psychiatrist when he comes out?'… 'no, I don't have that kind of access to psychiatrists'."*
> *GP1001, East*

Poor or delayed communication can result in misunderstandings about patients' support needs, and GP interviews highlighted that these high-risk patients could potentially suffer adverse events if their follow-up is not adequate. Hospitals and GPs communicate through discharge letters, which all GPs described as inadequate, often produced by junior doctors and lacking pertinent detail - "*the nuance, the detail is often missing*" (GP1005, North West).

Interviewees also reported examples of good practice, for example respiratory consultants sharing contact details and working closely with GPs when severe COVID-19 patients are discharged. In some cases, ICU interviewees commented on the importance of long-standing professional relationships with community rehabilitation service providers.

GPs were concerned about the evolving nature of COVID-19 and changing medical understanding. They welcomed specific and targeted information that would help them to guide patient's care after an intensive care and hospital stay. Others suggested a need for better communication with hospital teams to develop their understanding of specific patients' needs, and where to find support. One GP summarised the information needs as *"what to look for, when to refer back into hospital and types of patients that need specific follow-up" (GP1002, Yorkshire).*

All GPs stressed that guidance needs be balanced and channelled through a respected national body, as they faced an overload of information, particularly during the early phases of the pandemic when sometimes conflicting information was disseminated daily, from multiple sources.

To cope with the levels of information during the first wave, GP practices had initiated daily team meetings to discuss and keep abreast of key changes. One GP commented that the vast amount of COVID-19 information hampered GPs from employing their 'generalist skills' to tailor care to the individual's needs (GP1005, North West).

The pandemic as an opportunity to change

Interviewees from three ICUs described the pandemic as an opportunity to initiate a multidisciplinary team (MDT) follow-up clinic, by making visible the issues faced by patients discharged from ICU.

> *"[We've had an] uplift in the therapy staff […][as] we've now got more dietician[s], more physio, more pharmacy, we've never had an OT before six months ago, we've never had a psychologist of our own […] We're now in a position to offer MDT follow-up service rather than just a simple follow-up clinic."*
> *ICUcons06, South West.*

Interviewees highlighted the need for increased provision in response to the pandemic, resulting from large numbers of newly affected patients, uncertainties in their support needs, and a younger population needing to return to work.

For peer review only - http://bmjopen.bmj.com/site/about/guidelines.xhtml

Discussion

Statement of principal findings

The peak of the first wave of COVID-19 saw dramatic changes in ICUs to increase bed capacity. This was accompanied by adaptations to (and, in general, reductions in) the follow-up care provided, although most units retained some form of follow-up service.

Before COVID-19, there was a perception that funding streams and referral systems may hinder provision. The lack of a tariff for post-ICU follow-up may cause unwarranted variations, which ICU staff believe could be addressed through a 'reablement after critical care pathway' similar to that in place for cardiac and stroke rehabilitation.

Again, before the pandemic, communication between primary and secondary care was sometimes poor, and care was hampered by a lack of clarity about responsibilities for meeting various post-ICU patients' needs. GPs expressed a need for specific information about recovery from critical illness, collated by a single, authoritative professional group to avoid "*guideline fatigue*" (GP1004, London).

All of these existing constraints were believed to have been magnified by the COVID-19 pandemic.

Strengths and limitations of the study

To the best of our knowledge, this is the first study exploring NHS staff views on follow-up services post-ICU and plans to support patients recovering from severe COVID-19.

We cannot guarantee that our sample is representative of the UK. Responses to our ICU survey were spread across the country, cover different unit sizes, increases in capacity and sizes of NHS Trusts, and are similar to those reported by Connolly, et al. [14] GP responses to the survey and interviews was low but they were spread geographically, and all agreed on the challenges of organising care for patients discharged after an ICU stay. Our GP interview findings were consistent with each other, and similar to those with larger samples conducted before the pandemic.[15]

Meaning of the study: possible explanations and implications for clinicians and policymakers

A number of issues raised in this study are long standing: inadequate discharge summaries, lack of clarity of responsibility for post-acute patient care, fragmented and delayed communication and limited knowledge regarding the support needs of post-ICU patients. During the pandemic, there has been RCGP training about the main post-ICU sequelae, and potential treatments.[16 17] Problems in continuity of care, however, may need a joint approach to improve local organisation of care.

Community rehabilitation services were described as "patchy", with long waiting lists; an issue recognised by NHS England.[18] Recent initiatives to improve provision were welcomed, but some interviewees questioned whether the criteria for determining community rehabilitation needs were fit for purpose for younger, fitter populations, and whether community rehabilitation services could change provision without extra funding to enhance infrastructure. Community mental health services were particularly recognised as overstretched with long waiting lists that prioritise patients at high risk of harming themselves or others.[19] Murray, et al. [20] suggest a model such as the Nightingale hospital, but for rehabilitation, during and after the pandemic.

For peer review only - http://bmjopen.bmj.com/site/about/guidelines.xhtml

Commissioning and funding streams seem to be a major issue, as follow-up is recommended but not directly funded, unlike the pathways for cardiac and stroke rehabilitation, which were suggested by interviewees as models for post-ICU care. The evidence base for post-ICU follow-up is however partial and would benefit from further research. [21 22]

Our interviewees suggested that most of the long-term consequences faced by COVID-19 patients are similar to those faced by others experiencing ICU. Knowledge about the sequelae of COVID-19 is at an early stage, research on longer-term consequences of COVID-19, such as the Post-hospitalisation COVID-19 study (PHOSP-COVID) following more than 10,000 patients for more than 12 months, will shed light on which sequelae relate to being critically ill more generally and which are specific to COVID-19. This should complement what is already known about PICS and effective treatment models.[23]

One interviewee (Bruce J, personal communication) reported that patients who received CPAP reported worse mental health than patients ventilated invasively. According to the Intensive Care National Audit and Research Centre (ICNARC) report to the 9th of October, 44% of COVID-19 patients in critical care settings were not mechanically ventilated during the first 24 hours,[24] therefore a high proportion of patients are awake and aware of their surroundings. This is significant because, depending on the criteria for prioritisation, this group may not qualify for long-term follow-up, and consequently, might suffer from significant mental health symptoms without receiving formal support. Given the widespread management of COVID-19 patients with CPAP and high flow nasal cannulas, this cohort may need at least as much follow-up as those more invasively ventilated.

Unanswered questions and future research

Follow-up services vary greatly but the extent to which variations in provision are linked to differences in long-term outcomes is not clear. Identifying models of care which yield the best outcomes in the most efficient way could help develop the evidence base for reducing unwarranted variation in the future. The potential effect on mental health of receiving CPAP while in intensive care also merits further research.

Patients who have had an ICU stay might show impairments even five years after discharge. Currently, appropriate length of follow-up is unclear, as is the point at which care should be continued in primary and community care settings only. Current NICE guidance[25] addresses the early stage of follow-up, but not longer-term support.

The large cohort of younger than average ICU patients provides an opportunity to assess these services and ensure they meet the needs of those recovering from COVID-19 and other future patients discharged from intensive care.

For peer review only - http://bmjopen.bmj.com/site/about/guidelines.xhtml

Author contributions

This study was designed and conceived by ACA, LJ and KB. VD conducted the survey data analysis. ACA and LJ conducted the interviews and their analysis. ACA wrote the first draft of this manuscript. LJ and KB made comments on all the versions of this manuscript. All authors have read and agreed the final version.

Transparency statement

ACA affirms that this manuscript is an honest, accurate, and transparent account of the study being reported; that no important aspects of the study have been omitted; and that any discrepancies from the study as planned (and, if relevant, registered) have been explained.

Funding

This article is independent research commissioned and funded by the NIHR Policy Review Programme through the Partnership for Responsive Policy Analysis and Research (PREPARE), grant number NIHR 200702. The research was carried out between 15th April and 11th August. These findings were presented to key stakeholders in the Department of Health and Social Care. The funders had no role in considering the study design or in the collection, analysis, interpretation of data, writing of the report, or decision to submit the article for publication.

Disclaimer

The views expressed in this article are those of the authors and not necessarily those of the NIHR or the Department of Health and Social Care.

Competing interests

None declared

Data availability statement

Data from the interviews and survey responses are available upon reasonable request.

Dissemination to participants and related patient and public communities

The findings of the wider programme of research were shared with all our participants to check our interpretations captured their views. All the participants that replied confirmed our interpretations were accurate.

Ethics approval

This project was reviewed and approved by the Department of Health Ethics Committee at the University of York. ID Number HSRGC/2020/397/A.

Acknowledgments

We would like to acknowledge the contribution and advice of the Faculty of Intensive Care Medicine, the Intensive Care Society, the Royal College of General Practitioners, Dr Philip Anthill, Dr Shanthi Anthill, Prof Yvonne Birks, Dr Bronwen Connolly, Dr Tom Lawton, Prof Hugh Montgomery, Prof Rupert Pearse, Prof Zudin Puthucheary, Prof Trevor Sheldon, and Prof Najma Siddiqi.

For peer review only

For peer review only

References

1. Rengel KF, Hayhurst CJ, Pandharipande PP, et al. Long-term Cognitive and Functional Impairments After Critical Illness. *Anesthesia & Analgesia* 2019;128(4):772-80. doi: 10.1213/ane.0000000000004066
2. Desai SV, Law TJ, Needham DM. Long-term complications of critical care. *Critical Care Medicine* 2011;39(2):371-79. doi: 10.1097/CCM.0b013e3181fd66e5
3. Needham DM, Davidson J, Cohen H, et al. Improving long-term outcomes after discharge from intensive care unit: Report from a stakeholders' conference*. *Critical Care Medicine* 2012;40(2):502-09. doi: 10.1097/CCM.0b013e318232da75
4. Herridge MS, Tansey CM, Matté A, et al. Functional Disability 5 Years after Acute Respiratory Distress Syndrome. *New England Journal of Medicine* 2011;364(14):1293-304. doi: 10.1056/NEJMoa1011802
5. Van Aerde N, Meersseman P, Debaveye Y, et al. Five-year impact of ICU-acquired neuromuscular complications: a prospective, observational study. *Intensive Care Med* 2020;46(6):1184-93. doi: 10.1007/s00134-020-05927-5
6. Pfoh ER, Wozniak AW, Colantuoni E, et al. Physical declines occurring after hospital discharge in ARDS survivors: a 5-year longitudinal study. *Intensive Care Med* 2016;42(10):1557-66. doi: 10.1007/s00134-016-4530-1
7. Bienvenu OJ, Friedman LA, Colantuoni E, et al. Psychiatric symptoms after acute respiratory distress syndrome: a 5-year longitudinal study. *Intensive Care Med* 2018;44(1):38-47. doi: 10.1007/s00134-017-5009-4
8. Hodgson CL, Udy AA, Bailey M, et al. The impact of disability in survivors of critical illness. *Intensive Care Med* 2017;43(7):992-1001. doi: 10.1007/s00134-017-4830-0
9. McPeake J, Mikkelsen ME, Quasim T, et al. Return to Employment after Critical Illness and Its Association with Psychosocial Outcomes. A Systematic Review and Meta-Analysis. *Annals of the American Thoracic Society* 2019;16(10):1304-11. doi: 10.1513/AnnalsATS.201903-248OC
10. Hatch R, Young D, Barber V, et al. Anxiety, Depression and Post Traumatic Stress Disorder after critical illness: a UK-wide prospective cohort study. *Crit Care* 2018;22(1):310-10. doi: 10.1186/s13054-018-2223-6
11. FICM. Recovery and rehabilitation for patients following the Pandemic: Faculty of Intensive Care Medicine; 2020 [Available from: https://www.ficm.ac.uk/sites/default/files/ficm_rehab_provisional_guidance.pdf accessed 20 December 2020.
12. Wherton J, Greenhalgh T. Evaluation of the Attend Anywhere / Near Me video consulting service in Scotland, 2019-20. In: Government TS, ed. Social Research Series, 2020.
13. George PM, Barratt SL, Condliffe R, et al. Respiratory follow-up of patients with COVID-19 pneumonia. *Thorax* 2020;75(11):1009-16. doi: 10.1136/thoraxjnl-2020-215314
14. Connolly B, Douiri A, Steier J, et al. A UK survey of rehabilitation following critical illness: implementation of NICE Clinical Guidance 83 (CG83) following hospital discharge. *BMJ Open* 2014;4(5):e004963. doi: 10.1136/bmjopen-2014-004963
15. Bench S, Cornish J, Xyrichis A. Intensive care discharge summaries for general practice staff: a focus group study. *British Journal of General Practice* 2016;66(653):e904-e12. doi: 10.3399/bjgp16X688045
16. RCGP. COVID-19: The patient journey from rehabilitation to recovery: Royal College of General Practitioners; 2020 [Available from: https://elearning.rcgp.org.uk/mod/page/view.php?id=10822 accessed 26 Nov 2020.
17. RCGP. COVID-19: The Patient Journey Through Intensive Care: Royal College of General Practitioners; 2020 [Available from: https://elearning.rcgp.org.uk/mod/page/view.php?id=10801 accessed 26 Nov 2020.
18. NHS RightCare. RightCare: Community Rehabilitation Toolkit, 2020.

For peer review only - http://bmjopen.bmj.com/site/about/guidelines.xhtml

19. Naylor C, Bell A, Baird B, et al. Mental health and primary care networks: Understanding the opportunities: The King's Fund, 2020.
20. Murray A, Gerada C, Morris J. We need a Nightingale model for rehab after covid-19: HSJ; 2020 [Available from: https://www.hsj.co.uk/commissioning/we-need-a-nightingale-model-for-rehab-after-covid-19-/7027335.article accessed 9th October 2020.
21. Geense WW, van den Boogaard M, van der Hoeven JG, et al. Nonpharmacologic Interventions to Prevent or Mitigate Adverse Long-Term Outcomes Among ICU Survivors: A Systematic Review and Meta-Analysis*. *Critical Care Medicine* 2019;47(11):1607-18. doi: 10.1097/ccm.0000000000003974
22. McIlroy PA, King RS, Garrouste-Orgeas M, et al. The Effect of ICU Diaries on Psychological Outcomes and Quality of Life of Survivors of Critical Illness and Their Relatives: A Systematic Review and Meta-Analysis. *Critical Care Medicine* 2019;47(2):273-79. doi: 10.1097/ccm.0000000000003547
23. Waldmann CS. Intensive after care after intensive care. *Current Anaesthesia & Critical Care* 1998;9(3):134-39. doi: https://doi.org/10.1016/S0953-7112(98)80007-0
24. ICNARC. ICNARC report on COVID-19 in critical care: England, Wales and Northern Ireland, 9 October 2020. London, 2020.
25. NICE. Rehabilitation after Critical Illness. NICE Clinical Guideline 83. London, UK: National Institute for Health and Care Excellence, 2009.

For peer review only

For peer review only - http://bmjopen.bmj.com/site/about/guidelines.xhtml

Support and follow-up needs of patients discharged from Intensive Care after severe COVID-19: a mixed-methods study of the views of UK general practitioners and intensive care staff during the pandemic's first wave

Journal:	*BMJ Open*
Manuscript ID	bmjopen-2020-048392.R1
Article Type:	Original research
Date Submitted by the Author:	30-Mar-2021
Complete List of Authors:	Castro-Avila, Ana; University of York, Department of Health Sciences; Universidad del Desarrollo Facultad de Medicina Clínica Alemana, Carrera de Kinesiología<br>Jefferson, Laura; University of York, Department of Health Sciences<br>Dale, Veronica; University of York, Health Sciences<br>Bloor, Karen; University of York, Department of Health Sciences
**Primary Subject Heading**:	Health services research
Secondary Subject Heading:	Health services research, Qualitative research, General practice / Family practice, Intensive care
Keywords:	COVID-19, INTENSIVE & CRITICAL CARE, REHABILITATION MEDICINE

SCHOLARONE™ Manuscripts

I, the Submitting Author has the right to grant and does grant on behalf of all authors of the Work (as defined in the below author licence), an exclusive licence and/or a non-exclusive licence for contributions from authors who are: i) UK Crown employees; ii) where BMJ has agreed a CC-BY licence shall apply, and/or iii) in accordance with the terms applicable for US Federal Government officers or employees acting as part of their official duties; on a worldwide, perpetual, irrevocable, royalty-free basis to BMJ Publishing Group Ltd ("BMJ") its licensees and where the relevant Journal is co-owned by BMJ to the co-owners of the Journal, to publish the Work in this journal and any other BMJ products and to exploit all rights, as set out in our licence.

The Submitting Author accepts and understands that any supply made under these terms is made by BMJ to the Submitting Author unless you are acting as an employee on behalf of your employer or a postgraduate student of an affiliated institution which is paying any applicable article publishing charge ("APC") for Open Access articles. Where the Submitting Author wishes to make the Work available on an Open Access basis (and intends to pay the relevant APC), the terms of reuse of such Open Access shall be governed by a Creative Commons licence – details of these licences and which Creative Commons licence will apply to this Work are set out in our licence referred to above.

Other than as permitted in any relevant BMJ Author's Self Archiving Policies, I confirm this Work has not been accepted for publication elsewhere, is not being considered for publication elsewhere and does not duplicate material already published. I confirm all authors consent to publication of this Work and authorise the granting of this licence.

For peer review only

Support and follow-up needs of patients discharged from Intensive Care after severe COVID-19: a mixed-methods study of the views of UK general practitioners and intensive care staff during the pandemic's first wave

Ana Castro-Avila, Laura Jefferson, Veronica Dale, Karen Bloor

Department of Health Sciences, University of York, Heslington, YO10 5DD

Correspondence to A Castro-Avila ana.castro@york.ac.uk Area 4 Seebohm Rowntree Building, Department of Health Sciences, University of York, Heslington, YO10 5DD.

Keywords: Critical Illness/rehabilitation, Intensive care units, survivors, COVID-19

Word count: 4480

For peer review only

Abstract

Objectives: To identify follow-up services planned for COVID-19 patients discharged from ICU, and to explore the views of ICU staff and General Practitioners (GPs) regarding these patients' future needs and care coordination.

Design: Sequential mixed methods study using online surveys and semi—structured interviews. Interview data were inductively coded and thematically analysed. Survey data were descriptively analysed.

Setting: GPs surgeries and Acute NHS Trusts in the UK

Participants: GPs and clinicians leading care for patients discharged from ICU.

Primary and secondary outcomes: Usual follow-up practice after ICU discharge, changes in follow-up during the pandemic, and GP awareness of follow-up and support needs of patients discharged from ICU.

Results: We obtained 170 survey responses and conducted 23 interviews. Over 60% of GPs were unaware of the follow-up services generally provided by their local hospitals, and whether or not these were functioning during the pandemic. Eighty percent of ICUs reported some form of follow-up services, with 25% of these suspending provision during the peak of the pandemic, and over half modifying their provision (usually to provide the service remotely). Common themes relating to barriers to the provision of follow-up were funding complexities, remit and expertise, and communication between ICU and community services. Discharge documentation was described as poor and lacking key information. Both groups mentioned difficulties accessing services in the community and lack of clarity about who was responsible for referrals and follow-up.

Conclusions: The pandemic has highlighted long-standing issues of continuity of care and complex funding streams for post-ICU follow-up care. The large cohort of ICU patients admitted due to COVID-19 highlights the need for improved follow-up services and communication between specialists and GPs, not only for COVID-19 patients, but for all those discharged from ICU.

Article Summary

Strengths and limitations of this study

- This is the first study exploring NHS staff views of post-ICU follow-up services to support patients recovering from severe COVID-19.
- Responses to our survey had good geographic spread but were limited in number and relied on volunteers.
- GP interviews were small in number, but our findings align with those of larger studies conducted before the pandemic.

For peer review only - http://bmjopen.bmj.com/site/about/guidelines.xhtml

Introduction

The significant physical, mental and cognitive problems patients face following a stay in an Intensive Care Unit (ICU) are well-documented.[1 2] Including prolonged muscle weakness, cognitive dysfunction and symptoms of post-traumatic stress disorder (PTSD), these are often collectively referred to as 'post intensive care syndrome' (PICS)[3] and may persist even five years after leaving hospital.[4-7] Six months post-discharge, 25% of survivors suffer severe disability,[8] and only around 55% have returned to work.[9] Psychological disorders including depression, anxiety, and post-traumatic stress disorder (PTSD) are common, affecting 55% in the first year following ICU discharge.[10] The variety and severity of sequelae vary substantially.

The COVID-19 pandemic has resulted in a large and rapid increase in intensive care activity, which will challenge post-ICU services in several ways. Increases in ICU capacity necessitated the use of less experienced staff and very high workloads. More stringent infection control protocols created new potential causes of distress, including not allowing family members inside the unit and health care professionals wearing Personal Protective Equipment (PPE). These factors might lead to a very large cohort of critical illness survivors that might have greater than expected needs due to the context and length of their critical care stay, [11] which could put the current capacity of services under stress.

Post-ICU follow-up from hospital teams is likely to have been compromised during the pandemic. The availability, form and scale of services for ICU survivors of COVID-19 are largely unknown, and patient needs are difficult to predict. Poor communication and blurred lines of responsibility between secondary and primary care were identified as potential barriers for post-ICU care continuity before the pandemic.[12 13] Timely, appropriate support could potentially prevent future problems in patients' physical, cognitive and mental health and care; identifying how and to what extent these services have been scaled up is important to inform the future response of the health service. This study aims to identify follow-up services that were available during and after the UK's first wave of the COVID-19 pandemic, early reflections on care during the first wave and the views of critical care staff and General Practitioners (GPs) about these patients' future needs and care coordination.

Methods

We employed sequential mixed methods following a pragmatic approach. We collected data through online surveys and semi-structured telephone interviews with clinicians leading post-ICU follow-up and general practitioners (GPs).

In collaboration with clinicians in the field, we developed a very brief questionnaire of ICU staff to determine usual procedures of follow-up of patients after ICU discharge, and changes during the pandemic. The Intensive Care Society, Faculty of Intensive Care Medicine, British Association of Critical Care Nurses, and leading experts on intensive care disseminated this survey through newsletters, targeted emails and Twitter. We invited clinicians managing follow-up care of discharged ICU patients to participate, and asked respondents to volunteer for interviews. We sampled volunteers purposively by geographical location, and their responses to three of the survey questions: number of extra staffed beds opened during the pandemic, if they were offering follow-up services during the pandemic, and whether the provision of follow-up had changed.

In collaboration with the Royal College of General Practitioners (RCGP), we developed and distributed a very brief questionnaire exploring GPs' awareness of post-ICU follow-up services, and broad concerns about severe COVID-19 patients' care. RCGP also included three of these questions

For peer review only - http://bmjopen.bmj.com/site/about/guidelines.xhtml

in a routine survey of their GP research panel. GPs proved difficult to recruit to interviews through the survey, so we supplemented this with 'snowballing' using contacts at the RCGP, University of York and The King's Fund. We attempted to generate geographical spread in terms of location and COVID-19 incidence.

Both surveys were piloted with clinicians and experts. Initially, we shared the aim of the survey and the questions, so experts in the field could assess to what extent the questions provided the information needed, and language was precise, clear and concise. We then tested the survey with our target clinicians to ensure clarity and identify any essential omissions. We used Qualtrics XM software (Qualtrics, Provo, UT) to distribute the surveys and collect responses.

Mindful of time pressures for these health professionals at this time, we limited interviews to 30 minutes and designed topic guides to answer key questions around follow-up provision; aiming to generate more in-depth knowledge than could be achieved through survey alone. Interviews with ICU clinicians leading follow-up services explored views on whether and how the future needs of COVID-19 patients differed from non-COVID patients and captured early reflections on ICU care and transitions during the first wave of the COVID-19 outbreak. Interviews with GPs explored their prior experience of managing post-ICU patients, and information needs in relation to severe COVID-19. Potential participants were given Participant Information Leaflets and Consent Forms, detailing the ethical considerations (available as supplementary material) and verbal consent was obtained prior to each interview. The study was reviewed and approved by the University of York Department of Health Sciences Research Governance and Ethics Committee (ID Number HSRGC/2020/397/A).

Data analysis

Quantitative

Survey data were exported to IBM SPSS Statistics for Windows (Version 26. Armonk, NY: IBM Corp) for analysis. Absolute and relative frequencies were used to summarise responses. We calculated the average rank for the question about GPs' concerns regarding future care needs of patients recovering from a COVID-19 related critical care stay.

Qualitative

Interviews were audio-recorded and, as is increasingly being adopted in rapid qualitative research,[14] analysis was undertaken directly from audio-recordings and detailed notes. Sections were transcribed for use as quotations. We assigned a code and a number to each audio-recording and transcription to ensure anonymity.

An inductive approach was taken to analysing data using thematic analysis according to the six steps outlined by Braun and Clarke (2006, p35)[15] (Table 1). Following familiarisation with the data, two researchers (ACA and LJ) developed an initial coding framework of main themes, which were discussed with the wider team and topic experts to refine the framework and distil overarching themes. Some representative quotes are presented to contextualise and aid interpretation.

A reflexive approach was taken during the analysis process to consider how the researchers' presence and a priori assumptions may have influenced the data collection and analysis stages. The lead researcher (ACA) is a trained ICU physiotherapist, which influenced the research question chosen and could have affected the themes that are presented in this article. Through regular discussion amongst the research team as themes were developed and refined, we limited any impact of researcher bias on the process of data analysis.

For peer review only - http://bmjopen.bmj.com/site/about/guidelines.xhtml

Table 1 Stages of qualitative thematic analysis based on Braun and Clarke (2006).

Phases of thematic analysis	Description
1. Familiarisation with the data	Interview notes were made immediately following each interview and were reviewed multiple times, along with audio recordings.
2. Generating initial codes	Initial codes were developed into a framework through discussion between the two primary coders (ACC and LJ), with wider team members and topic experts. Data, in the form of interview notes with transcribed sections, was sorted using data management software (NVivo 12, QSR International Pty Ltd). The same coding framework was applied across both participant groups: GPs and ICU.
3. Searching for themes	Initial codes were categorised into overarching themes, drawing out similarities and differences across participant groups. The nine initial major themes and 21 subthemes were revised in line with the aims of this research.
4. Reviewing themes	Themes were reviewed and refined by the study team. Overlaps were reorganised into higher order themes. For example, 'communication' appeared as a challenge for direct patient care, delivering news to family members, and ensuring continuity of care outside of the hospital. Therefore, communication was reorganised into a higher order theme comprising these three different aspects.
5. Defining and naming themes	A final framework of four themes and three subthemes was developed.
6. Producing the report	Qualitative and survey findings were integrated, combining the more descriptive qualitative findings with those from the survey that pertain to the ICU environment and provision. More 'thematic' qualitative findings were summarised separately. Participant quotes were used to illustrate key points and provide transparency in this process.

Patient and Public Involvement (PPI)

We reviewed and discussed the project protocol with our PPI group, refining it in response. We were, however, unable to capture patients' views on follow-up services in our study timescale.

For peer review only - http://bmjopen.bmj.com/site/about/guidelines.xhtml

Results

Descriptive findings

Between 15th June and 3rd August, ICU follow-up lead clinicians from 112 units (43% of Acute NHS Trusts in England) responded to our survey. 83% were based in England, and 96% were from mixed intensive care and high dependency units. On average, units more than doubled bed capacity at the height of the first wave (Table 2).

Follow-up services were offered in 80 units (71% of those sampled); of those, 20 reported ceasing provision and 53 modifying provision of services during the pandemic. Eight units implemented a new follow-up service after the peak of the pandemic. Provision of occupational therapy and physiotherapy were the services with the greatest increase (Table 2).

For peer review only

For peer review only - http://bmjopen.bmj.com/site/about/guidelines.xhtml

Table 2 Responses of ICU leads about follow-up services during the pandemic

Information about the unit	n=112	Responses		
When is the critical care discharge summary sent to the patient's General Practitioner?	38	When the patient is discharged from hospital		
	36	After critical care discharge, but before discharge from hospital		
	16	Other		
	8	I do not know		
When is the first follow-up?	29	2-3 months after discharge from hospital		
	28	2-3 months after discharge from critical care		
	11	Other		
	8	4-6 months after discharge from hospital		
	3	1 month after discharge from hospital		
Number of beds in your unit, mean (SD)	93	Before: 13.9 (11.1)<br>During peak: 33.7 (31.0)<br>Change: 20.1 (23.9)		
**Changes reported**	**n=53**	**Details of change**		
Change in the format of the contacts (e.g. remote consultations)	39	Remote consultations via telephone or video call		
	15	Face-to-face clinics in hospital wearing personal protective equipment		
	2	Home visits		
Change in the number of professionals involved, mean (SD)	22	Before: 2.8 (1.9)<br>After: 4.1 (2.4)<br>Change: 1.3 (2.6)		
Change in the timing of the first contact	24	Time before first follow-up contact is shorter than usual		
	2	Time before first follow-up contact is longer than usual		
Services available	25		Before	During
		Review of ICU history/diary and ICU events with patient	23	22
		Assessment of sleep	15	12
		Physiotherapy	13	17
		Medicines reconciliation	10	8
		Psychology	9	10
		Assessment of sexual function	8	4
		Dietetics	6	5
		Speech and Language Therapy	5	6
		Cognitive assessment,	5	4
		Psychiatry,	2	2
		Social work	2	0
		Occupational Therapy,	1	5

For peer review only - http://bmjopen.bmj.com/site/about/guidelines.xhtml

Table 3 Responses from GPs survey

Question	Responses	Our sample	RCGP
Does your nearest Hospital Trust have specific follow-up services for all patients who have been discharged from critical care?	n	58	-
	I do not know	36 (62%)	-
	Yes	11 (19%)	-
	No	11 (19%)	-
Is the follow-up service functioning during the COVID-19 pandemic?	n	45	-
	I do not know	39 (87%)	-
	Yes	6 (13%)	-
Within your patient list, are you aware of any patients who have required critical care for severe COVID-19?	n	56	537
	Yes	33 (59%)	208 (39%)
	No	13 (23%)	244 (45%)
	I do not know	10 (18%)	85 (16%)
How many of your patients went through critical care due to severe COVID-19?	n	24	462
	Mean (min-max)	4.4 (1-20)	4.4 (0-50)
Considering future patients in your practice recovering from a COVID-19 related critical care stay, please rank your concerns about their care, mean rank (SD)	n	40	447
	Physical health care	1.9 (1.4)	1.4 (1.3)
	Mental Health care	2.4 (1.1)	1.4 (1.2)
	Access to rehabilitation services	3.1 (1.3)	1.6 (1.3)
	Cognitive functioning	3.5 (1.2)	1.8 (1.3)
	Access to social care	4.0 (1.1)	1.7 (1.4)

58 GPs responded to our survey and an additional 537 responded to three questions we distributed via the RCGP (Table 3). 78% of RCGP responses came from England, 61% were female, 63% were 35-54 years old, and 83% were white. Over 60% of GPs were unaware of the follow-up services generally provided by their local hospitals, and whether or not these were functioning during the pandemic. On average, 4 patients from their patient's list had been through ICU due to severe COVID-19. Physical and mental health care needs were ranked similarly high in terms of areas of concern with future patients recovering after a critical care stay.

We conducted 23 interviews between 23rd June and 30th July; 17 with ICU staff (7 ICU consultants, 7 senior nurses, 3 rehabilitation coordinators) and 6 GPs. ICU interviews covered all UK regions, with the ICUs having an average capacity before the pandemic of 14 beds (range 4-60), increasing by 16 beds on average (range 2-38 beds). The GPs covered different regions of England and a mix of patient demographics.

Since the aim of our interviews was predominantly to provide a more in-depth descriptive account of current ICU provision, much of these findings are summarised descriptively under the headings 'ICU environment' and 'provision of services'. More thematic findings are summarised under the themes 'barriers' and 'opportunity for change.'

The ICU environment during the COVID-19 first wave

All interviewees reported opening new areas and bringing nurses from other areas (e.g. theatre, surgical recovery, other hospital wards) particularly those with ICU training. Consultants increased the frequency of their rotations to ensure continuous coverage. Administrative tasks for clinicians were suspended and all staff providing outpatient or outreach services returned to inpatient activities. ICU nurses split their time between patient care, staff supervision and training new staff, which was reported to increase workload and stress. Hospitals with greater bed capacity

For peer review only

implemented proning and intubating teams, and some implemented retrieval teams to transfer patients between hospitals.

Numerous ICU interviewees mentioned that patients with COVID-19 may represent a new patient group, but are still ICU survivors, with the weakness, mental and cognitive problems these patients commonly suffer. They expected COVID-19 patients to suffer a longer-lasting deterioration of lung function, potential issues with renal function, a high incidence of shoulder injuries due to proning, and cognitive problems related to the incidence of delirium.

Some thought it was too early to tell whether they will experience more physiological and psychological problems, but many highlighted particular treatments, including prolonged and deep sedation, opioids and neuromuscular blockers, which are associated with increased risk of muscular weakness, polyneuropathy, and cognitive impairments. Patients experienced extended periods in a prone position, mechanical ventilation and less experienced nursing staff. One consultant believed that actively screening for mental health problems was needed (ICUcons09, Scotland).

One ICU nurse (ICUnurse04), who regularly administers mental health questionnaires to ICU patients, had observed results from those with COVID-19. She reported that the ventilated patients had similar psychological issues as pre-COVID-19 ICU patients, but those who received continuous positive airways pressure (CPAP), and were therefore conscious, had worse scores. An ICU consultant echoed this and also highlighted potential difficulties due to PPE:

> *"The other people in the bay watched [another patient] die over a number of days… It doesn't surprise me that the people here perhaps, more awake and aware are very, very traumatised by the experiences […] [Some patients] have delusional thoughts. I mean, I think that's gonna be a lot worse when you're surrounded by someone wearing a hazmat suit." ICUcons06, South West.*

Provision of follow-up services

Before COVID-19, most ICU interviewees reported having a post-ICU follow-up service; the few that did not were planning to implement one after the pandemic. Most follow-up services were suspended during the peak of the first wave, as staff returned to in-hospital clinical duties. The few places that continued to provide such services used telephone follow-up, delivered by staff that were shielding.

Reported provision varies greatly, with some units delivering follow-up with just a consultant and/or a senior nurse, while others have multidisciplinary teams. Some units start their follow-up during the ICU stay, and have designated professionals to assess, refer and follow patients during the hospital episode and into the community. Others with well-established follow-up services refer ICU patients to pulmonary or cardiac rehabilitation services to recover fitness and muscle strength.

All unit staff we interviewed follow patients up 2-3 months after ICU discharge, but a minority also routinely call patients weekly (ICUnurse04, North East) or monthly (ICUnurse08, East Midlands). All had to change the format of their follow-up during the pandemic, most replaced clinics with telephone calls or virtual consultations. One senior nurse highlighted the challenges of these virtual contacts due to reduced non-verbal communication and time limits:

> *"Phone calls don't really cut it because unless you're very skilled at talking to people, assessing people, you're not going to pick up on all those cues that people*

For peer review only - http://bmjopen.bmj.com/site/about/guidelines.xhtml

> *give out […] if we've got half an hour appointment, we won't get much out from in 10 minutes, but they'll open up" ICUnurse14, East of England*

Two ICU interviewees said that they were implementing separate clinics for COVID-19 patients to carry out extra recommended assessments, such as a chest x-ray at 6 weeks post-discharge as recommended by the British Thoracic Society.[16]

In some locations COVID-19 rehab hospitals have been set up to provide specialist care and a "step down" for patients *"that are not quite well enough to leave the acute setting and not quite well enough to go home"* (GP1003, Yorkshire). This provided the opportunity for expert care to be delivered but relied on CCG funding and *"proactive planning for the worst-case scenario"* (GP1003, Yorkshire).

GPs were concerned about the complex psychological needs of patients recovering from severe COVID-19 and that greater emphasis is placed on the physical needs of patients, with insufficient consideration of psychological support. All ICU interviewees agreed about the need for increased psychological support services.

Some ICU interviewees questioned the capacity of community rehabilitation services to improve patients' functioning. Both GPs and ICU staff felt that previous notions of thresholds for functional status post-ICU (several commented on assessments of patients climbing a flight of stairs) were arbitrary and not suitable for the wider age group of patients affected by COVID-19:

> *"Community Services work at getting someone functional. They don't work at getting them back to the state that they were at before they came into hospital. So, considering that a lot of our patients were younger patients, walking with a Zimmer frame to and from a bathroom aren't really what they want to be doing. They want to be getting back to their fitness level and back to work." ICUrehab15, Wales*

One GP commented that: *"it's only once they are home that the true level of need is understood"* (GP1005, North West); at this point primary care and community services need to step in, but the support needs of these patients may be beyond their expertise and the capacity of services.

Some GP practices had taken proactive steps to follow-up patients discharged from ICU (before and since COVID-19). One practice, pre-COVID-19, developed a list of 'at risk' patients that was monitored by a nurse practitioner and discussed at daily practice meetings, with the aim of reducing hospital admissions. One London practice had instigated weekly follow-up calls with COVID-19 patients discharged from ICU, following a 'near miss' event whereby a serious complication had been detected opportunistically during a GP follow-up call.

Thematic findings

Barriers to provision of follow-up services

Interviewees commented on various components that acted as barriers to the provision of follow-up services relating predominantly to funding complexities, remit and expertise, and communication between ICU and community services.

For peer review only - http://bmjopen.bmj.com/site/about/guidelines.xhtml

Funding complexities

ICU interviewees in England felt the lack of a tariff for funding ICU follow-up clinics created variation in service provision. Both ICU and GP interviewees believed that community teams similar to those for stroke or cardiac rehabilitation should be set up for post-ICU patients.

Several interviewees were concerned that already overstretched community services with existing waiting lists could not meet the increase in demand from COVID-19 without improved funding and infrastructure. One GP described the closure of some community services, and ICU interviewees had concerns that those discharged from ICU did not have anywhere to go.

> *"We've had patients in tears, we've had seven patients through telephone calls. And all of them are absolutely distraught and feel like they've been abandoned in the community [...] because they were thrown out of hospital very quickly. There's no services in the community for them at all." ICUrehab15, Wales*

Remit and expertise

Both ICU staff and GPs found referrals to community follow-up services difficult, with differences in opinion about whose responsibility this was, as well as problems with waiting lists (particularly for mental health services). Community rehabilitation services were described as *"patchy"* (ICUnurse08, East Midlands). Both staff groups felt that hospital services were better placed to follow-up patients discharged from ICU, because they have a better understanding of the patient's needs. There appears, however, to be a general lack of awareness about the difficulties of coordinating patients' needs in each setting.

> *"I'm not sure the hospitals are always very aware of what services are available in the community… To give you a COVID example, I had a doctor ring me up and say 'he's been in hospital for a long time, can you make sure he sees a psychiatrist when he comes out?'… 'no, I don't have that kind of access to psychiatrists'." GP1001, East*

Communication

Poor or delayed communication can result in misunderstandings about patients' support needs, and GP interviews highlighted that these high-risk patients could potentially suffer adverse events if their follow-up is not adequate. Hospitals and GPs communicate through discharge letters, which all GPs described as inadequate, often produced by junior doctors and lacking pertinent detail - "*the nuance, the detail is often missing*" (GP1005, North West).

Interviewees also reported examples of good practice, for example respiratory consultants sharing contact details and working closely with GPs when severe COVID-19 patients are discharged. In some cases, ICU interviewees commented on the importance of long-standing professional relationships with community rehabilitation service providers.

GPs were concerned about the evolving nature of COVID-19 and changing medical understanding. They welcomed specific and targeted information that would help them to guide patient's care after an intensive care and hospital stay. Others suggested a need for better communication with hospital teams to develop their understanding of specific patients' needs, and where to find support. One GP summarised the information needs as *"what to look for, when to refer back into hospital and types of patients that need specific follow-up" (GP1002, Yorkshire).*

For peer review only

All GPs stressed that guidance needs be balanced and channelled through a respected national body, as they faced an overload of information (described by one as "*guideline fatigue*" (GP1004, London). This was particularly cumbersome during the early phases of the pandemic when sometimes conflicting information was disseminated daily, from multiple sources.

To cope with the levels of information during the first wave, some GP practices had initiated daily team meetings to discuss and keep abreast of key changes. One GP commented that the vast amount of COVID-19 information hampered GPs from employing their '*generalist skills'* to tailor care to the individual's needs (GP1005, North West).

The pandemic as an opportunity to change

Interviewees from three ICUs described the pandemic as an opportunity to initiate a multidisciplinary team (MDT) follow-up clinic, by making visible the issues faced by patients discharged from ICU.

> *"[We've had an] uplift in the therapy staff [...][as] we've now got more dietician[s], more physio, more pharmacy, we've never had an OT before six months ago, we've never had a psychologist of our own [...] We're now in a position to offer MDT follow-up service rather than just a simple follow-up clinic." ICUcons06, South West.*

Interviewees highlighted the need for increased provision in response to the pandemic, resulting from large numbers of newly affected patients, uncertainties in their support needs, and a younger population needing to return to work.

Discussion

Statement of principal findings

The peak of the first wave of COVID-19 saw dramatic changes in ICUs to increase bed capacity. This was accompanied by adaptations to (and, in general, reductions in) the follow-up care provided, although most units retained some form of follow-up service.

Before COVID-19, there was a perception that funding streams and referral systems may hinder provision. The lack of a tariff for post-ICU follow-up may cause unwarranted variations, which interviewees believed could be addressed through a 'reablement after critical care pathway' similar to that in place for cardiac and stroke rehabilitation.

Again, before the pandemic, communication between primary and secondary care was sometimes poor, and care was hampered by a lack of clarity about responsibilities for meeting various post-ICU patients' needs. GPs expressed a need for specific information about recovery from critical illness, collated by a single, authoritative professional group.

All of these existing constraints were believed to have been magnified by the COVID-19 pandemic.

Strengths and limitations of the study

To the best of our knowledge, this is the first study exploring NHS staff views on follow-up services post-ICU and plans to support patients recovering from severe COVID-19.

For peer review only - http://bmjopen.bmj.com/site/about/guidelines.xhtml

Our recruitment strategy relied heavily on social media due to time constraints, which might have attracted participants that are more willing to share their opinions. Potentially, a different recruitment approach might have yielded different results. While we cannot guarantee that our survey samples are representative of the UK, responses were spread across the country, covering different ICU unit sizes, increases in capacity and sizes of NHS Trusts, and are similar to those reported by Connolly, et al. [17] who used a different recruitment strategy and included a larger sample. GP responses to the survey were low but they were spread geographically, and had similar views to the larger sample from the RCGP survey. GPs tend to see very few patients that have been discharged from intensive care,[12] and it is not clear how this might affect our results considering that participants were self-selected.

While our qualitative interviews did not seek to achieve generalisability, our GP interview findings were consistent with each other, and similar to those with larger samples conducted before the pandemic.[12 13] This study formed part of a larger project and was conducted rapidly to inform UK health policy during the peak of the pandemic. Nevertheless, our qualitative methods followed principles considered to promote rigour in qualitative research. Investigator and methodological triangulation methods were employed to develop rich and in-depth understanding; findings are evidenced using quotations to enhance the transparency and trustworthiness of conclusions drawn; findings were shared with our interviewees to ensure that our interpretations were accurate; and a reflexive approach was adopted to consider how the researchers' a priori assumptions may have affected data collection and analysis.

Meaning of the study: possible explanations and implications for clinicians and policymakers

A number of issues raised in this study are long standing and have been highlighted in previous research:[12 13] inadequate discharge summaries, lack of clarity of responsibility for post-acute patient care, fragmented and delayed communication and limited knowledge regarding the support needs of post-ICU patients. During the pandemic, there has been RCGP training about the main post-ICU sequelae and potential treatments,[18 19] which could help to improve awareness around the mental, physical, and cognitive consequences of an ICU stay. Problems in continuity of care, however, may need a joint approach to improve local organisation of care and how information is delivered across settings considering a "whole patient journey".[13] Discharge summaries written by more senior staff in hospital highlighting potential red flags and greatest awareness in secondary care regarding the capabilities of primary care were suggested as elements that could improve the communication and transition between secondary and primary care.

Commissioning and funding streams seem to be a major issue, as follow-up is recommended but not directly funded, unlike the pathways for cardiac and stroke rehabilitation, which were suggested by interviewees as models for post-ICU care. The evidence base for post-ICU follow-up is however partial and would benefit from further research. [20 21] Interviewees highlighted that the specialised nature of post-ICU care meant that intensive care staff were better placed to understand and refer patient to services for cognitive, physical and mental health problems, but funding did not always allow this. Additionally, interviewees suggested that services that were required for longer such as talking therapies and physical rehabilitation should be delivered in the community, where they might be more easily accessed.

Community rehabilitation services were described as "patchy", with long waiting lists; an issue recognised by NHS England.[22] Recent initiatives to improve provision were welcomed, but some

For peer review only - http://bmjopen.bmj.com/site/about/guidelines.xhtml

interviewees questioned whether the criteria for determining community rehabilitation needs were fit for purpose for younger, fitter populations, and whether community rehabilitation services could change provision without extra funding to enhance infrastructure. Community mental health services were particularly recognised as overstretched with long waiting lists that prioritise patients at high risk of harming themselves or others.[23] Murray, et al. [24] suggest a model such as the Nightingale hospital, but for rehabilitation, during and after the pandemic.

Our interviewees suggested that most of the long-term consequences faced by COVID-19 patients are similar to those faced by others experiencing ICU. Knowledge about the sequelae of COVID-19 is at an early stage, research on longer-term consequences of COVID-19, such as the Post-hospitalisation COVID-19 study (PHOSP-COVID) following more than 10,000 patients for more than 12 months, will shed light on which sequelae relate to being critically ill more generally and which are specific to COVID-19. This should complement what is already known about PICS and effective treatment models.[25]

One interviewee mentioned that patients who received CPAP reported worse mental health than patients ventilated invasively. While this was only reported by one interviewee and should therefore be interpreted with caution, it may warrant further exploration in wider samples or research as, according to the Intensive Care National Audit and Research Centre (ICNARC) report to the 9th of October 2020, 44% of COVID-19 patients in critical care settings were not mechanically ventilated during the first 24 hours.[26] This implies that a high proportion of patients are awake and aware of their surroundings. Depending on the criteria for prioritisation, they may not qualify for long-term follow-up, and consequently, might suffer from mental health symptoms without receiving formal support. Given the widespread management of COVID-19 patients with CPAP and high flow nasal cannulas, this cohort may need at least as much follow-up as those ventilated more invasively.

Unanswered questions and future research

Follow-up services vary greatly but the extent to which variations in provision are linked to differences in long-term outcomes is not clear. Identifying models of care which yield the best outcomes in the most efficient way could help develop the evidence base for reducing unwarranted variations in the future. The potential effect on mental health of being in intensive care while receiving CPAP may merit further research.

Patients who have had an ICU stay might show impairments even five years after discharge. Currently, appropriate length of follow-up is unclear, as is the point at which care should be continued in primary and community care settings only. Current NICE guidance[27] addresses the early stage of follow-up, but not longer-term support.

The large cohort of younger than average ICU patients provides an opportunity to assess these services and ensure they meet the needs of those recovering from COVID-19 and other future patients discharged from intensive care.

For peer review only

Author contributions

This study was designed and conceived by ACA, LJ and KB. VD conducted the survey data analysis. ACA and LJ conducted the interviews and their analysis. ACA wrote the first draft of this manuscript. LJ and KB made comments on all the versions of this manuscript. All authors have read and agreed the final version.

Transparency statement

ACA affirms that this manuscript is an honest, accurate, and transparent account of the study being reported; that no important aspects of the study have been omitted; and that any discrepancies from the study as planned (and, if relevant, registered) have been explained.

Funding

This article is independent research commissioned and funded by the NIHR Policy Review Programme through the Partnership for Responsive Policy Analysis and Research (PREPARE), grant number NIHR 200702. The research was carried out between 15th April and 11th August. These findings were presented to key stakeholders in the Department of Health and Social Care. The funders had no role in considering the study design or in the collection, analysis, interpretation of data, writing of the report, or decision to submit the article for publication.

Disclaimer

The views expressed in this article are those of the authors and not necessarily those of the NIHR or the Department of Health and Social Care.

Competing interests

None declared

Data availability statement

Data from the interviews and survey responses are available upon reasonable request.

Dissemination to participants and related patient and public communities

The findings of the wider programme of research were shared with all our participants to check our interpretations captured their views. All the participants that replied confirmed our interpretations were accurate.

Ethics approval

This project was reviewed and approved by the Department of Health Ethics Committee at the University of York. ID Number HSRGC/2020/397/A.

Acknowledgments

We would like to acknowledge the contribution and advice of the Faculty of Intensive Care Medicine, the Intensive Care Society, the Royal College of General Practitioners, Dr Philip Antill, Dr Shanthi Antill, Prof Yvonne Birks, Dr Bronwen Connolly, Dr Tom Lawton, Prof Hugh Montgomery, Prof Rupert Pearse, Prof Zudin Puthucheary, Prof Trevor Sheldon, and Prof Najma Siddiqi.

For peer review only - http://bmjopen.bmj.com/site/about/guidelines.xhtml

For peer review only

For peer review only - http://bmjopen.bmj.com/site/about/guidelines.xhtml

References

1. Rengel KF, Hayhurst CJ, Pandharipande PP, et al. Long-term Cognitive and Functional Impairments After Critical Illness. *Anesthesia & Analgesia* 2019;128(4):772-80. doi: 10.1213/ane.0000000000004066
2. Desai SV, Law TJ, Needham DM. Long-term complications of critical care. *Critical Care Medicine* 2011;39(2):371-79. doi: 10.1097/CCM.0b013e3181fd66e5
3. Needham DM, Davidson J, Cohen H, et al. Improving long-term outcomes after discharge from intensive care unit: Report from a stakeholders' conference*. *Critical Care Medicine* 2012;40(2):502-09. doi: 10.1097/CCM.0b013e318232da75
4. Herridge MS, Tansey CM, Matté A, et al. Functional Disability 5 Years after Acute Respiratory Distress Syndrome. *New England Journal of Medicine* 2011;364(14):1293-304. doi: 10.1056/NEJMoa1011802
5. Van Aerde N, Meersseman P, Debaveye Y, et al. Five-year impact of ICU-acquired neuromuscular complications: a prospective, observational study. *Intensive Care Med* 2020;46(6):1184-93. doi: 10.1007/s00134-020-05927-5
6. Pfoh ER, Wozniak AW, Colantuoni E, et al. Physical declines occurring after hospital discharge in ARDS survivors: a 5-year longitudinal study. *Intensive Care Med* 2016;42(10):1557-66. doi: 10.1007/s00134-016-4530-1
7. Bienvenu OJ, Friedman LA, Colantuoni E, et al. Psychiatric symptoms after acute respiratory distress syndrome: a 5-year longitudinal study. *Intensive Care Med* 2018;44(1):38-47. doi: 10.1007/s00134-017-5009-4
8. Hodgson CL, Udy AA, Bailey M, et al. The impact of disability in survivors of critical illness. *Intensive Care Med* 2017;43(7):992-1001. doi: 10.1007/s00134-017-4830-0
9. McPeake J, Mikkelsen ME, Quasim T, et al. Return to Employment after Critical Illness and Its Association with Psychosocial Outcomes. A Systematic Review and Meta-Analysis. *Annals of the American Thoracic Society* 2019;16(10):1304-11. doi: 10.1513/AnnalsATS.201903-248OC
10. Hatch R, Young D, Barber V, et al. Anxiety, Depression and Post Traumatic Stress Disorder after critical illness: a UK-wide prospective cohort study. *Crit Care* 2018;22(1):310-10. doi: 10.1186/s13054-018-2223-6
11. FICM. Recovery and rehabilitation for patients following the Pandemic: Faculty of Intensive Care Medicine; 2020 [Available from: https://www.ficm.ac.uk/sites/default/files/ficm_rehab_provisional_guidance.pdf accessed 20 December 2020.
12. Bench S, Cornish J, Xyrichis A. Intensive care discharge summaries for general practice staff: a focus group study. *British Journal of General Practice* 2016;66(653):e904-e12. doi: 10.3399/bjgp16X688045
13. Zilahi G, O'Connor E. Information sharing between intensive care and primary care after an episode of critical illness; A mixed methods analysis. *PLOS ONE* 2019;14(2):e0212438. doi: 10.1371/journal.pone.0212438
14. Wherton J, Greenhalgh T. Evaluation of the Attend Anywhere / Near Me video consulting service in Scotland, 2019-20. In: Government TS, ed. Social Research Series, 2020.
15. Braun V, Clarke V. Using thematic analysis in psychology. *Qualitative Research in Psychology* 2006;3(2):77-101. doi: 10.1191/1478088706qp063oa
16. George PM, Barratt SL, Condliffe R, et al. Respiratory follow-up of patients with COVID-19 pneumonia. *Thorax* 2020;75(11):1009-16. doi: 10.1136/thoraxjnl-2020-215314
17. Connolly B, Douiri A, Steier J, et al. A UK survey of rehabilitation following critical illness: implementation of NICE Clinical Guidance 83 (CG83) following hospital discharge. *BMJ Open* 2014;4(5):e004963. doi: 10.1136/bmjopen-2014-004963
18. RCGP. COVID-19: The patient journey from rehabilitation to recovery: Royal College of General Practitioners; 2020 [Available from: https://elearning.rcgp.org.uk/mod/page/view.php?id=10822 accessed 26 Nov 2020.

For peer review only - http://bmjopen.bmj.com/site/about/guidelines.xhtml

19. RCGP. COVID-19: The Patient Journey Through Intensive Care: Royal College of General Practitioners; 2020 [Available from: https://elearning.rcgp.org.uk/mod/page/view.php?id=10801 accessed 26 Nov 2020.
20. Geense WW, van den Boogaard M, van der Hoeven JG, et al. Nonpharmacologic Interventions to Prevent or Mitigate Adverse Long-Term Outcomes Among ICU Survivors: A Systematic Review and Meta-Analysis*. *Critical Care Medicine* 2019;47(11):1607-18. doi: 10.1097/ccm.0000000000003974
21. McIlroy PA, King RS, Garrouste-Orgeas M, et al. The Effect of ICU Diaries on Psychological Outcomes and Quality of Life of Survivors of Critical Illness and Their Relatives: A Systematic Review and Meta-Analysis. *Critical Care Medicine* 2019;47(2):273-79. doi: 10.1097/ccm.0000000000003547
22. NHS RightCare. RightCare: Community Rehabilitation Toolkit, 2020.
23. Naylor C, Bell A, Baird B, et al. Mental health and primary care networks: Understanding the opportunities: The King's Fund, 2020.
24. Murray A, Gerada C, Morris J. We need a Nightingale model for rehab after covid-19: HSJ; 2020 [Available from: https://www.hsj.co.uk/commissioning/we-need-a-nightingale-model-for-rehab-after-covid-19-/7027335.article accessed 9th October 2020.
25. Waldmann CS. Intensive after care after intensive care. *Current Anaesthesia & Critical Care* 1998;9(3):134-39. doi: https://doi.org/10.1016/S0953-7112(98)80007-0
26. ICNARC. ICNARC report on COVID-19 in critical care: England, Wales and Northern Ireland, 9 October 2020. London, 2020.
27. NICE. Rehabilitation after Critical Illness. NICE Clinical Guideline 83. London, UK: National Institute for Health and Care Excellence, 2009.

For peer review only

Supplementary material

Version 1.0 (12/05/2020)

THE UNIVERSITY of York

Post-ICU support for patients discharged after COVID 19: Participant Information Sheet

We would like to invite you to take part in a research study. Before you decide, it is important for you to understand why this research is being done and what it would involve for you. Please take time to read the following information carefully and discuss it with others if you wish. Ask us if there is anything that is not clear or if you would like more information. Take time to decide whether or not you wish to take part.

Thank you for reading this.

What is the purpose of the study?

Evidence shows that patients discharged following an Intensive Care Unit (ICU) stay may experience significant physical, mental and cognitive problems after leaving the unit and once they return to their homes and communities. These may persist even five years after leaving hospital[1] and a recent study shows that six months post-discharge, 25% of survivors suffer severe disability, 22% have depression or anxiety and 40% have returned to work.[2]

The COVID-19 pandemic represents clear challenges, with follow-up and transition post-ICU stay likely to have been unavoidably curtailed. This research project is part of a wider study looking at the availability and form of follow-up services available for ICU survivors of COVID-19. This interview study will ask a sample of ICU leads and critical care staff about their views around the future needs of patients post-ICU discharge following severe COVID illness and early reflections on ICU care and transitions out of ICU during the COVID-19 outbreak.

Why am I being asked to take part?

As part of the project we are interviewing ICU leads and critical care staff to explore views and experiences around the needs of COVID patients, post-ICU discharge. You have been selected to represent one of these perspectives and we are keen to talk to you more about this.

Do I have to take part?

No, it is entirely up to you to decide whether you would like to take part. If you have any questions about taking part you can talk to a member of the research team. Even if you have agreed to take part, you are free to withdraw from the study at any time, without giving a reason.

What will happen to me if I take part?

If you decide that you would like to take part in the study you will be sent a consent form electronically to read, which will be discussed prior to interview. We will then ask you to give verbal consent during the telephone or videoconference interview.

A researcher will approach you to arrange a time to conduct the interview that best suits you. Due to the current need for social distancing, interviews will take place remotely either by telephone or videoconference call. The interview will last up around 30 minutes and will be audio-recorded (with your permission), and sections transcribed for analysis.

---

[1] Herridge et al (2011) Functional Disability 5 Years after Acute Respiratory Distress Syndrome. N Engl J Med; 364:1293-1304. DOI: 10.1056/NEJMoa1011802
[2] Hodgson et al (2017) The impact of disability in survivors of critical illness. Intensive Care Med.;43(7):992-1001. DOI: 10.1007/s00134-017-4830-0

Version 1.0 (12/05/2020)

You will be given a unique study number for the duration of the study so that your name and organisation will not be used in any publications and will not be made available outside the research team.

What are the possible benefits of taking part?

Whilst there are no personal benefits to you for taking part, the findings of this study will develop a better understanding of the future needs of COVID 19 patients discharged from ICU.

Expenses and payments

This study is funded by the NIHR Policy Research Programme and we will arrange interviews at a time convenient to you so that no costs will be associated with you taking part in the study. Thus, no participant expenses have been allocated for this study.

What will happen to data that are collected about me?

We will remove all names and other identifying information before the data are analysed and results presented. Any records that identify you will be held separately to the other information we collect and your data will be held in a secure location, in accordance with the General Data Protection Regulations (GDPR) and the Data Protection Act 2018. Only researchers that are part of the research team in York and The King's Fund will have access to the data. Data will be stored for 5 years, to enable analysis and publication and will then be destroyed. If you decide to change your mind about taking part in the study, you can request that the data collected be destroyed. Following this, your data will not be analysed or used in the report of the findings.

Who has reviewed this study?

This study has been reviewed by the University of York's Department of Health Sciences' Research Governance Committee.

Who is organising and funding this research?

The research is funded by the NIHR Policy Research Programme. The research funding covers only the costs of undertaking the research; researchers will not receive payment for conducting the study. Findings will be reported in aggregated form, interviewees and organisations will not be named or otherwise identifiable when findings are reported.

Who can I contact for more information?

If you have any queries about the study please contact:<br><br>Dr Laura Jefferson<br>Research Fellow<br>Department of Health Sciences<br>Area 4 Seebohm Rowntree Building<br>University of York<br>Heslington<br>York YO10 5DD<br><br>Email: laura.jefferson@york.ac.uk		If you need to make a complaint or speak to someone independent, please contact:<br><br>Prof Patrick Doherty<br>Chair of Research Committee<br>Department of Health Sciences<br>Area 4 Seebohm Rowntree Building<br>University of York<br>Heslington<br>York<br><br>Email: patrick.doherty@york.ac.uk

Thank you for reading this information sheet and for considering whether to take part in this study.

Version 1.0 (12/05/2020)

THE UNIVERSITY of York

CONSENT FORM

Participant Identification Number: ☐☐☐☐

Title of study: Post-ICU support for patients discharged after COVID 19: interviews with critical care staff

	**Please initial the boxes to confirm verbal consent given**
1. I confirm that I have read and understand the information sheet version [1] dated [12/05/20] for the above study. I have had the opportunity to consider the information, ask questions and have had these answered satisfactorily.	☐
2. I understand that my participation is voluntary and that I am free to withdraw at any time without giving reason.	☐
3. I agree to this consent form and other data collected as part of this research study being kept at the University of York and The King's Fund.	☐
4. I understand that relevant sections of data collected during the study may be looked at by individuals from the University of York and The King's Fund and from regulatory authorities. I give permission for these individuals to have access to these records.	☐
6. I agree to the interviews being audio recorded and sections transcribed.	☐
7. I understand that direct quotations may be used in publications but no information will be released or printed that would identify me.	☐
8. I understand and agree that the research team will securely store my identifiable details in order to contact me in future regarding this study, or other related studies (e.g. telephone/text/email). Identifiable details, including a copy of the consent form, will be available only to the research team, other than for purposes of monitoring and audit.	☐
9. I agree to take part in the above study.	☐

____________________ ____________________

Name of participant *(please print)* Date verbal consent given

____________________ ____________________

Name of person taking consent *(please print)* Date

Version 1.0 (29/04/2020)

THE UNIVERSITY of York

Post-ICU support for patients discharged after COVID 19: Participant Information Sheet

We would like to invite you to take part in a research study. Before you decide, it is important for you to understand why this research is being done and what it would involve for you. Please take time to read the following information carefully and discuss it with others if you wish. Ask us if there is anything that is not clear or if you would like more information. Take time to decide whether or not you wish to take part.

Thank you for reading this.

What is the purpose of the study?

Evidence shows that patients discharged following an Intensive Care Unit (ICU) stay may experience significant physical, mental and social problems after leaving the unit and once they return to their homes and communities. These may persist even five years after leaving hospital[1] and a recent study shows that six months post-discharge, 25% of survivors suffer severe disability, 22% have depression or anxiety and 40% have returned to work.[2]

The COVID-19 pandemic represents clear challenges, with follow-up and transition post-ICU stay likely to have been unavoidably curtailed. This research project is part of a wider study looking at the availability and form of follow-up services available for ICU survivors of COVID-19. This interview study will explore GPs experience of managing patients following an ICU stay in general and those patients discharged following severe COVID illness. We will also identify any information needs amongst GPs in relation to patients recovering from severe COVID illness.

Why am I being asked to take part?

As part of the project we are interviewing GPs to explore how patients are managed in general practice following discharge from ICU, in general and following severe COVID illness. You have been selected to represent one of these perspectives and we are keen to talk to you more about this.

Do I have to take part?

No, it is entirely up to you to decide whether you would like to take part. If you have any questions about taking part, you can talk to a member of the research team. Even if you have agreed to take part, you are free to withdraw from the study at any time, without giving a reason.

What will happen to me if I take part?

If you decide that you would like to take part in the study you will be sent a consent form electronically to read, which will be discussed prior to interview. We will then ask you to give verbal consent during the telephone or videoconference interview.

A researcher will approach you to arrange a time to conduct the interview that best suits you. Due to current social distancing restrictions, interviews will take place remotely either by telephone or videoconference call. The interview will last up around 30 minutes and will be used to find out more information about your experiences of managing patients following usual

---

[1] Herridge et al (2011) Functional Disability 5 Years after Acute Respiratory Distress Syndrome. N Engl J Med; 364:1293-1304. DOI: 10.1056/NEJMoa1011802
[2] Hodgson et al (2017) The impact of disability in survivors of critical illness. Intensive Care Med.;43(7):992-1001. DOI: 10.1007/s00134-017-4830-0

Version 1.0 (29/04/2020)

discharge from ICU, and discharge following severe COVID illness. The interview will be audio-recorded, with your permission, and sections may be transcribed for analysis.

You will be given a unique study number for the duration of the study so that your name and organisation will not be used in any publications and will not be made available outside the research team.

What are the possible benefits of taking part?

Whilst there are no personal benefits to you for taking part, the findings of this study will develop a better understanding of the practices being used to care for COVID 19 patients discharged from ICU and any informational needs within general practice.

Expenses and payments

This study is funded by the NIHR Policy Research Programme and we will arrange interviews at a time convenient to you so that no costs will be associated with you taking part in the study. Thus, no participant expenses have been allocated for this study.

What will happen to data that are collected about me?

We will remove all names and other identifying information before the data are analysed and results presented. Any records that identify you will be held separately to the other information we collect, and your data will be held in a secure location, in accordance with the General Data Protection Regulations (GDPR) and the Data Protection Act 2018. Only researchers that are part of the research team in York and The King's Fund will have access to the data. Data will be stored for 5 years, to enable analysis and publication and will then be destroyed. If you decide to change your mind about taking part in the study, you can request that the data collected be destroyed. Following this, your data will not be analysed or used in the report of the findings.

Who has reviewed this study?

This study has been reviewed by the University of York's Department of Health Sciences' Research Governance Committee.

Who is organising and funding this research?

The research is funded by the NIHR Policy Research Programme. The research funding covers only the costs of undertaking the research; researchers will not receive payment for conducting the study. Findings will be reported in aggregated form, interviewees and organisations will not be named or otherwise identifiable when findings are reported.

Who can I contact for more information?

If you have any queries about the study please contact:<br><br>Dr Laura Jefferson<br>Research Fellow<br>Department of Health Sciences<br>Area 4 Seebohm Rowntree Building<br>University of York<br>Heslington<br>York YO10 5DD<br><br>Email: laura.jefferson@york.ac.uk		If you need to make a complaint or speak to someone independent, please contact:<br><br>Prof Patrick Doherty<br>Chair of Research Committee<br>Department of Health Sciences<br>Area 4 Seebohm Rowntree Building<br>University of York<br>Heslington<br>York<br><br>Email: patrick.doherty@york.ac.uk

Thank you for reading this information sheet and for considering whether to take part in this study.

Version 1.0 (29/04/2020)

THE UNIVERSITY of York

CONSENT FORM

Participant Identification Number: ☐☐☐☐

Title of study: Post-ICU support for patients discharged after COVID 19: interviews with GPs

Please initial the boxes to confirm verbal consent given

1. I confirm that I have read and understand the information sheet version [1] dated [29/04/20] for the above study. I have had the opportunity to consider the information, ask questions and have had these answered satisfactorily. ☐

2. I understand that my participation is voluntary and that I am free to withdraw at any time without giving reason. ☐

3. I agree to this consent form and other data collected as part of this research study being kept at the University of York and The King's Fund. ☐

4. I understand that relevant sections of data collected during the study may be looked at by individuals from the University of York and The King's Fund and from regulatory authorities. I give permission for these individuals to have access to these records. ☐

6. I agree to the interviews being audio recorded and sections transcribed. ☐

7. I understand that direct quotations may be used in publications but no information will be released or printed that would identify me. ☐

8. I understand and agree that the research team will securely store my identifiable details in order to contact me in future regarding this study, or other related studies (e.g. telephone/text/email). Identifiable details, including a copy of the consent form, will be available only to the research team, other than for purposes of monitoring and audit. ☐

9. I agree to take part in the above study. ☐

____________________ ____________________

Name of participant *(please print)* Date verbal consent given

____________________ ____________________

Name of person taking consent *(please print)* Date

Standards for Reporting Qualitative Research (SRQR) Checklist

No.	Topic	Item	Page
	**Title and abstract**		
S1	Title	Concise description of the nature and topic of the study Identifying the study as qualitative or indicating the approach (e.g., ethnography, grounded theory) or data collection methods (e.g., interview, focus group) is recommended	1
S2	Abstract	Summary of key elements of the study using the abstract format of the intended publication; typically includes background, purpose, methods, results, and conclusions	2
	**Introduction**		
S3	Problem formulation	Description and significance of the problem/phenomenon studied; review of relevant theory and empirical work; problem statement	3
S4	Purpose or research question	Purpose of the study and specific objectives or questions	3
	**Methods**		
S5	Qualitative approach and research paradigm	Qualitative approach (e.g., ethnography, grounded theory, case study, phenomenology, narrative research) and guiding theory if appropriate; identifying the research paradigm (e.g., postpositivist, constructivist/ interpretivist) is also recommended; rationale [b]	3
S6	Researcher characteristics and reflexivity	Researchers' characteristics that may influence the research, including personal attributes, qualifications/experience, relationship with participants, assumptions, and/or presuppositions; potential or actual interaction between researchers' characteristics and the research questions, approach, methods, results, and/or transferability	4
S7	Context	Setting/site and salient contextual factors; rationale [b]	7-9
S8	Sampling strategy	How and why research participants, documents, or events were selected; criteria for deciding when no further sampling was necessary (e.g., sampling saturation); rationale [b]	3
S9	Ethical issues pertaining to human subjects	Documentation of approval by an appropriate ethics review board and participant consent, or explanation for lack thereof; other confidentiality and data security issues	S1
S10	Data collection methods	Types of data collected; details of data collection procedures including (as appropriate) start and stop dates of data collection and analysis, iterative process, triangulation of sources/methods, and modification of procedures in response to evolving study findings; rationale [b]	4-5
S11	Data collection instruments and technologies	Description of instruments (e.g., interview guides, questionnaires) and devices (e.g., audio recorders) used for data collection; if/how the instrument(s) changed over the course of the study	4
S12	Units of study	Number and relevant characteristics of participants, documents, or events included in the study; level of participation (could be reported in results)	5
S13	Data processing	Methods for processing data prior to and during analysis, including transcription, data entry, data management and security, verification of data integrity, data coding, and anonymization/deidentification of excerpts	4
S14	Data analysis	Process by which inferences, themes, etc., were identified and developed, including the researchers involved in data analysis; usually references a specific paradigm or approach; rationale [b]	4-5
S15	Techniques to enhance trustworthiness	Techniques to enhance trustworthiness and credibility of data analysis (e.g., member checking, audit trail, triangulation); rationale [b]	4-5
	**Results/findings**		
S16	Synthesis and interpretation	Main findings (e.g., interpretations, inferences, and themes); might include development of a theory or model, or integration with prior research or theory	7-11
S17	Links to empirical data	Evidence (e.g., quotes, field notes, text excerpts, photographs) to substantiate analytic findings	8-11
	**Discussion**		
S18	Integration with prior work, implications, transferability, and contribution(s) to the field	Short summary of main findings; explanation of how findings and conclusions connect to, support, elaborate on, or challenge conclusions of earlier scholarship; discussion of scope of application/ generalizability; identification of unique contribution(s) to scholarship in a discipline or field	11
S19	Limitations	Trustworthiness and limitations of findings	11-12
	**Other**		
S20	Conflicts of interest	Potential sources of influence or perceived influence on study conduct and conclusions; how these were managed	14
S21	Funding	Sources of funding and other support; role of funders in data collection, interpretation, and reporting	14

[a] The authors created the SRQR by searching the literature to identify guidelines, reporting standards, and critical appraisal criteria for qualitative research; reviewing the reference lists of retrieved sources; and contacting experts to gain feedback. The SRQR aims to improve the transparency of all aspects of qualitative research by providing clear standards for reporting qualitative research. [b] The rationale should briefly discuss the justification for choosing that theory, approach, method, or technique rather than other options available, the assumptions and limitations implicit in those choices, and how those choices influence study conclusions and transferability. As appropriate, the rationale for several items might be discussed together.

For peer review only
